# The BMAL1/HIF2A heterodimer modulates circadian variations of myocardial injury

**DOI:** 10.21203/rs.3.rs-3938716/v1

**Published:** 2024-02-28

**Authors:** Wei Ruan, Tao Li, Jaewoong Lee, In Hyuk Bang, Wankun Deng, Xinxin Ma, Seung-Hee Yoo, Boyun Kim, Jiwen Li, Xiaoyi Yuan, Yu A An, Yin-Ying Wang, Yafen Liang, Matthew Deberge, Dongze Zhang, Zhen Zhou, Yanyu Wang, Josh Gorham, Jonathan G. Seidman, Christine E. Seidman, Sary F. Aranki, Ragini Nair, Lei Li, Jagat Narula, Zhongming Zhao, Alemayehu G Abebe, Jochen Daniel Muehlschlegel, Kuang-Lei Tsai, Holger K. Eltzschig

**Affiliations:** 1 Department of Anesthesiology, Critical Care and Pain Medicine, The University of Texas Health Science Center at Houston, McGovern Medical School, Houston, TX, 77030, USA.; 2 Department of Anesthesiology, Second Xiangya Hospital, Central South University, Changsha, 410011, China.; 3 Department of Biochemistry and Molecular Biology, The University of Texas Health Science Center at Houston, McGovern Medical School, Houston, TX, 77030, USA.; 4 Department of Anesthesiology, Yale University School of Medicine, New Haven, CT, 06510, USA.; 5 Center for Precision Health, McWilliams School of Biomedical Informatics, The University of Texas Health Science Center at Houston, Houston, TX, 77030, USA.; 6 Department of Cardiac Surgery, Sir Run Run Shaw Hospital, School of Medicine, Zhejiang University, Hangzhou, 310016, China.; 7 Division of Medical Genetics, Department of Internal Medicine, The University of Texas Health Science Center at Houston, McGovern Medical School, Houston, TX, 77030, USA.; 8 Department of Genetics, Harvard Medical School, Boston, MA, 02115, USA.; 9 Department of Surgery, Division of Cardiac Surgery, Brigham and Women’s Hospital, Harvard Medical School, Boston, MA, 02115, USA.; 10 Institute of Systems and Physical Biology, Shenzhen Bay Laboratory, Shenzhen, 518055, China.; 11 Weatherhead PET Center for Preventing and Reversing Atherosclerosis, Division of Cardiology, Department of Medicine, The University of Texas Health Science Center at Houston, McGovern Medical School, Memorial Hermann Hospital, Houston, TX, 77030, USA.; 12 Department of Integrative Biology and Pharmacology, The University of Texas Health Science Center at Houston, McGovern Medical School, Houston, TX, 77030, USA.; 13 Department of Anesthesiology and Critical Care Medicine, Johns Hopkins Medicine, Baltimore, MD, 21287, USA.; 14 MD Anderson Cancer Center, UTHealth Houston Graduate School of Biomedical Sciences, Houston, TX, USA.; 15 Outcomes Research Consortium, Cleveland, OH, USA.; 16 These authors contributed equally.

## Abstract

Acute myocardial infarction stands as a prominent cause of morbidity and mortality worldwide^1–6^. Clinical studies have demonstrated that the severity of cardiac injury following myocardial infarction exhibits a circadian pattern, with larger infarct sizes and poorer outcomes in patients experiencing morning onset myocardial infarctions^7–14^. However, the molecular mechanisms that govern circadian variations of myocardial injury remain unclear. Here, we show that BMAL1^14–20^, a core circadian transcription factor, orchestrates diurnal variability in myocardial injury. Unexpectedly, BMAL1 modulates circadian-dependent cardiac injury by forming a transcriptionally active heterodimer with a non-canonical partner, hypoxia-inducible factor 2 alpha (HIF2A)^6,21–23^, in a diurnal manner. Substantiating this finding, we determined the cryo-EM structure of the BMAL1/HIF2A/DNA complex, revealing a previously unknown capacity for structural rearrangement within BMAL1, which enables the crosstalk between circadian rhythms and hypoxia signaling. Furthermore, we identified amphiregulin (AREG) as a rhythmic transcriptional target of the BMAL1/HIF2A heterodimer, critical for regulating circadian variations of myocardial injury. Finally, pharmacologically targeting the BMAL1/HIF2A-AREG pathway provides effective cardioprotection, with maximum efficacy when aligned with the pathway’s circadian trough. Our findings not only uncover a novel mechanism governing the circadian variations of myocardial injury but also pave the way for innovative circadian-based treatment strategies, potentially shifting current treatment paradigms for myocardial infarction.

Acute myocardial infarction (MI) remains a leading cause of morbidity and mortality worldwide^1–6,23,24^. Clinical studies have demonstrated a circadian pattern in MI outcomes, with larger infarct sizes and a higher incidence of heart failure in patients with morning-onset MIs^7–14^. Despite this well-established diurnal pattern, current cardioprotective strategies have yet to fully integrate these insights^25–31^, resulting in less effective treatments and, in some cases, even failures, mainly due to a limited understanding of the underlying mechanisms. To address these challenges, we hypothesize that exploring and leveraging endogenous cardioprotective mechanisms during circadian phases of lesser injury might be key to developing more effective treatment strategies.

Circadian rhythms synchronize internal biological functions with environmental changes across diverse organisms, facilitating adaptation to the daily fluctuations stemming from the Earth’s day-night cycles^32–35^. Central to these rhythms are core transcription factors, notably brain and muscle Arnt-like 1 (BMAL1)^14–20^, which typically heterodimerizes with CLOCK, initiating the transcription of clock-controlled genes by binding to E-boxes in their promoter regions. While clinical and pre-clinical studies have highlighted the importance of circadian rhythms in cardiovascular physiology and disease^13,14,31,36^, the specific regulatory mechanisms governing circadian variations of myocardial injury remain largely unexplored.

Here, we identified BMAL1 as a pivotal transcription factor in modulating diurnal variations in myocardial injury through transcriptomic profiling using left ventricular tissues from both mice and human patients experiencing myocardial injury at different times of the day. Importantly, we demonstrated that cardiomyocyte-specific deletion of *Bmal1* eliminates inherent circadian variability in myocardial injury and diminishes endogenous cardioprotection. Unexpectedly, in contrast to its canonical partner, CLOCK^19,20,37,38^, we identified hypoxia-inducible factor 2 alpha (HIF2A)^6,21–23^ as a non-canonical and rhythmic interacting partner of BMAL1 during MI. Cardiomyocyte-specific deletion of *Hif2a* mirrored the dampening of cardioprotection observed in *Bmal1*-deficent mice. Furthermore, we discovered that *AREG* is a crucial rhythmic target of the BMAL1/HIF2A complex, and mice lacking *Areg* exhibited similar phenotypes, showing abolished circadian-dependent cardioprotection. Timed AREG administration or enhancing BMAL1’s activity substantially improved cardioprotection, particularly when synchronized with their circadian trough.

Collectively, we unveiled a novel molecular mechanism that integrates the intricate crosstalk between circadian rhythms and hypoxia signaling into cardioprotective strategies, offering valuable insights for optimizing current approaches and pioneering clock-based therapies for MI. Additionally, by elucidating the first structure of the BMAL1/HIF2A/DNA complex, we uncovered a previously unknown capability of BMAL1 to undergo structural rearrangement upon binding with distinct partners in response to circadian rhythms and hypoxia signaling. With these two pathways being universal and essential in almost all cells and organs, our findings have far-reaching implications for understanding and treating a wide spectrum of ischemic diseases exhibiting circadian patterns.

## Identification of BMAL1 as a key transcription factor in circadian variations of myocardial injury

**Extended Data Fig. 1.**
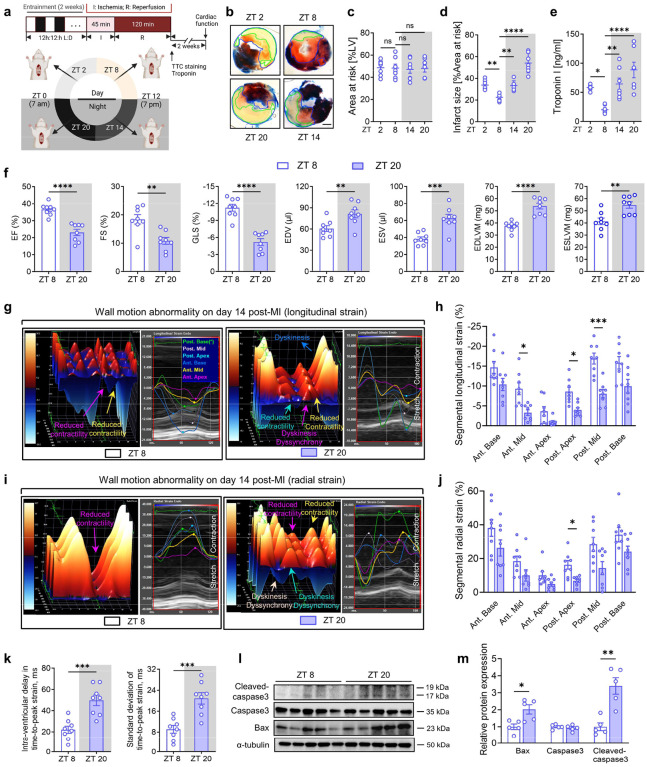
Circadian variation of myocardial injury in C57BL/6J mice. **a,** Schematic of the experimental setup for evaluating myocardial injury and cardiac function in C57BL/6J mice subjected to myocardial IRI at different ZTs (ZT2, ZT8, ZT14, and ZT20). **b-e, b,** Representative heart slices subjected to Evan’s blue and TTC double staining following 2 h of reperfusion: infarct area (green line) and area at risk (AAR; blue line); scale bar, 1 mm. **c,** Percentage of the AAR relative to the size of LV. **d,** Infarct sizes represented as the percentage of the AAR. **e,** Serum troponin I level. b-c: n = 7 mice/group/time point. Statistical analysis was performed using one-way ANOVA. **f-k,** Cardiac function was measured by speckle-tracking echocardiography analysis on day 14 post-MI in C57BL/6J mice subjected to myocardial IRI at ZT8 or ZT20. **f,** Left ventricular systolic function in ejection fraction (EF), fractional shortening (FS), and global longitudinal strain (GLS); left ventricular end-diastolic volume (EDV) and end-systolic volume (ESV); end-diastolic left ventricular mass (EDLVM) and end-systolic left ventricular mass (ESLVM). Statistical analysis was performed using unpaired Student’s *t*-tests. **g,** Representative left ventricular 3D longitudinal strain (48 points) and 6-segment longitudinal strain images demonstrating wall motion abnormalities (Color-coded six segments: Ant. Base, Anterior Base; Ant. Mid, Anterior Middle; Ant. Apex, Anterior Apex; Post. Apex, Posterior Apex; Post. Mid, Posterior Middle; Post. Base, Posterior Base). **h,** Left ventricular segmental wall contractility detected by peak longitudinal strain. Statistical analysis was performed using unpaired two-way ANOVA. **i,** Representative left ventricular 3D long-axis radial strain (48 points) and 6-segment radial strain images demonstrating wall motion abnormalities. **j,** Left ventricular segmental wall contractility detected by peak radial strain. Statistical analysis was performed using unpaired two-way ANOVA. **k,** Left ventricular mechanical dyssynchrony as measured by intra-ventricular delay in time-to-peak strain and standard deviation of time-to-peak strain based on peak longitudinal strain. Statistical analysis was performed using unpaired Student’s *t*-tests. f-k, n = 7 mice/group/time point. **l,** Protein levels of cleaved-caspase3, caspase3, and Bax were measured by Western blot analysis in the AAR of mouse hearts on day 3 post-MI. **m,** Quantification of protein levels in **(l)**. n = 5 mice/group/time point. Statistical analysis was performed using unpaired two-way ANOVA. All data are mean ± s.e.m, *p < 0.05, **p < 0.01, ***p < 0.001, and ****p < 0.0001, ns: not significant.

To elucidate the mechanisms underlying circadian variation of myocardial injury, we adopted a previously established murine model showing circadian-dependent cardiac injury following MI^5^. Myocardial injury and cardiac function were evaluated at the acute phase (2h of reperfusion) and the extended reperfusion periods (14 days). Specifically, C57BL/6J mice were subjected to myocardial ischemia and reperfusion injury (IRI) via left coronary artery ligation for 45 minutes followed by reperfusion at Zeitgeber Time (ZT) 2, ZT8, ZT14, and ZT20 under standard 12 h light/12 h dark entrainment conditions (Extended Data Fig. 1a). Consistent with previous studies^5,39,40^, we observed diurnal variations of myocardial injury, with the smallest infarctions at ZT8 and the most severe injury at ZT20 following 2h of reperfusion, as shown by infarct sizes and serum troponin I levels (Extended Data Fig. 1b–e). To determine whether these diurnal variations of myocardial injury observed after 2h of reperfusion corresponded to differential long-term outcomes, we evaluated cardiac function on day 14 post-MI using speckle-tracking echocardiography (STE). We chose STE for its adeptness in the early detection of subtle cardiac changes^41^, and its capacity for detailed and reproducible assessment of global and regional LV function and remodeling post-MI^42–44^. In support of our short-term findings, mice subjected to IRI at ZT20 exhibited a pronounced deterioration in LV systolic function, a significant increase in LV size and mass, more severe segmental wall motion abnormalities^45^, and a notable increase in intra-ventricular dyssynchrony^46–49^ (Extended Data Fig. 1f–k). Furthermore, we noticed a marked increase in apoptosis in the ZT20 mouse hearts relative to the ZT8 group on day 3 post-MI, as indicated by elevated expressions of cleaved-caspase3 and Bax (Extended Data Fig. 1l,m). These findings indicate that, consistent with clinical research, the timing of myocardial IRI can profoundly influence the severity of the injury, resulting in significant long-term structural and functional changes to the heart.

**Extended Data Fig. 2.**
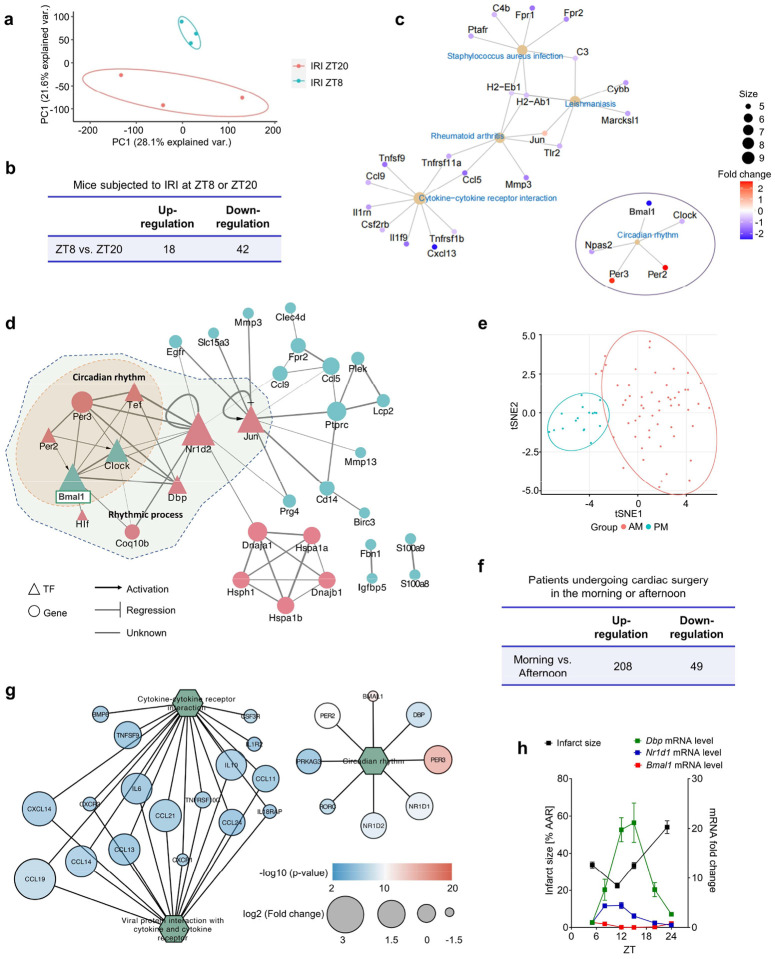
BMAL1 is a key transcription factor in regulating circadian variation of myocardial injury. **a-d,** RNA-seq analysis was conducted on the AAR from C57BL/6J mice subjected to myocardial IRI at ZT8 or ZT20. **a,** PCA of mRNA expression profiles. **b,** DEGs were detected in mouse hearts when comparing ZT8 vs. ZT20 (fold change > 1.5, adjusted p < 0.05). **c,** The top five enriched KEGG pathways for DEGs in the ZT8 vs. ZT20 groups. **d,** Gene dysregulation network constructed using DEGs. Triangle represents transcription factor (TF), and circle denotes gene. Green nodes represent downregulated TF/genes, while red nodes denote upregulated TF/genes in the ZT8 mouse hearts compared to the ZT20 group. The size of the node represents the degree in the network, and the width of the edges represents the strength of correlations. n =3 mice/group/time point. **e-g,** Human RNA-seq analysis using left ventricular biopsies from cardiac surgical patients in the morning (AM) or afternoon (PM) groups. **e,** PCA demonstrating the distinct transcriptional signatures across different patient groups. **f,** DEGs when comparing AM with PM patient samples (log2 fold change > 0.5 and p < 0.01). **g,** The top three KEGG pathways enriched by DEGs. The colors and size of nodes represent the p value and fold change of each gene. n = 56/morning and n = 17/afternoon. **h,** The peak transcriptional activity of BMAL1 in the ischemic mouse hearts, demonstrated by the maximum expression of its target genes (*Dbp* and *Nr1d1*) at ZT8, aligns with the least myocardial injury. Data modified from Fig. 1c and Extended Data Fig. 2d. n = 7/infarct size measurement and n = 3/gene expression analysis. All data are mean ± s.e.m.

**Fig. 1.**
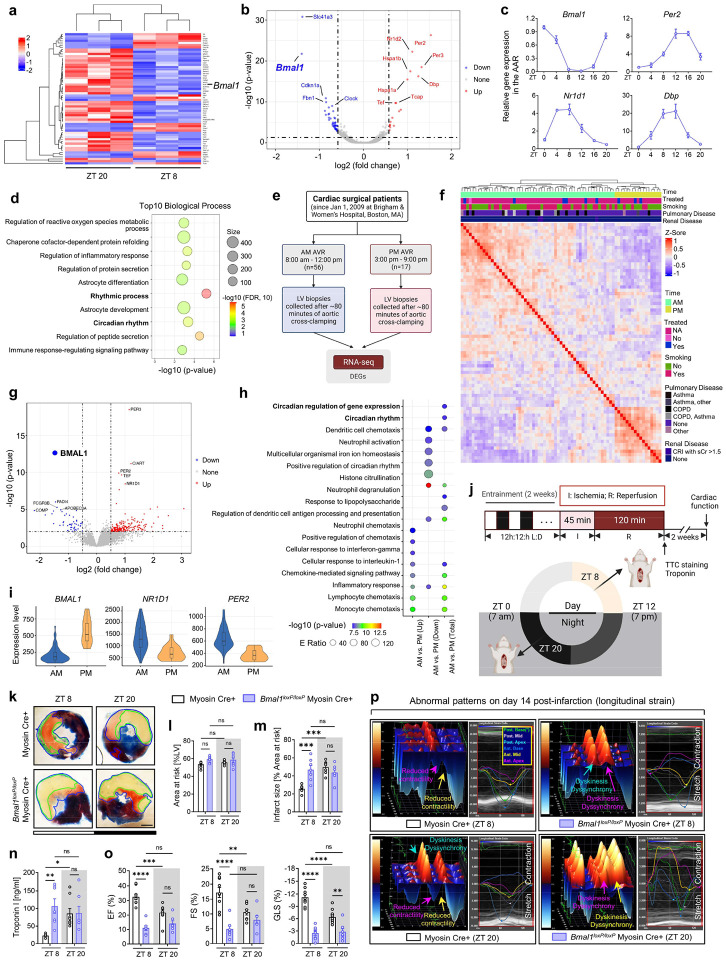
Identification of BMAL1 in modulating the circadian variation of myocardial injury. **a,** Heatmap with expression patterns of DEGs in the AAR from mouse hearts subjected to myocardial IRI at ZT8 or ZT20. n = 3 mice/time point. **b,** Volcano plot showing DEGs when comparing ZT8 to ZT20. n = 3 mice/time point. **c,** Circadian expression patterns of *Bmal1* and its target genes, *Per2*, *Nr1d1*, and *Dbp*, by real-time PCR analysis in the AAR of mouse hearts, subjected to IRI at different times of the day (ZT0, ZT4, ZT8, ZT12, ZT16, and ZT20). n = 3 mice/time point. **d,** Top 10 enriched GO biological process terms in IRI mouse hearts. **e,** Schematic illustration of human RNA-seq analysis using LV biopsies from cardiac surgical patients in the morning (AM) or afternoon (PM) groups. n = 56/morning and n = 17/afternoon. **f,** Heatmap showing clustered Pearson’s correlation matrix of patient LV samples. Major covariates, including treatment status, smoking history, pulmonary disease, and renal disease, were shown at the top. **g,** Volcano plot showing DEGs when comparing AM to PM patient LV samples. **h,** Enriched GO biological process terms for upregulated, down-regulated, and differentially expressed genes. **i,** Normalized read count for *BMAL1*, *NR1D1*, and *PER2* in patient LV samples. **j,** Experimental setup for evaluation of myocardial injury and cardiac function in *Bmal1*^*loxP/loxP*^ Myosin Cre+ mice and Myosin Cre+ mice subjected to IRI at ZT8 or ZT20. **k,** Representative heart slices subjected to Evan’s blue and TTC double staining after 2 h of reperfusion: infarct area (green line) and AAR (blue line); scale bar, 1 mm. **l,** Percentage of the AAR relative to the size of the LV. n = 7 mice/group/time point. Statistical analysis was performed using two-way ANOVA. **m,** Infarct sizes represented as the percentage of the AAR. n = same number as above. Statistical analysis was performed using two-way ANOVA. **n,** Serum troponin I levels were evaluated at 2h after reperfusion. n = 7/*Bmal1*^*loxP/loxP*^ Myosin Cre+ mice/time point and n = 8/Myosin Cre+/time point. Statistical analysis was performed using two-way ANOVA. **o and p,** Cardiac function was evaluated by speckle-tracking echocardiography on day 14 post-MI. **o,** LV systolic function in EF, FS, and GLS. n = 7/*Bmal1*^*loxP/loxP*^ Myosin Cre+ mice/time point and n = 9/Myosin Cre+ mice/time point. Statistical analysis was performed using two-way ANOVA. **p,** Representative left ventricular 3D (48 points) longitudinal strain and 6-segment longitudinal strain images demonstrating wall motion abnormalities (Color-coded six segments: Ant. Base, Anterior Base; Ant. Mid, Anterior Middle; Ant. Apex, Anterior Apex; Post. Apex, Posterior Apex; Post. Mid, Posterior Middle; Post. Base, Posterior Base). All data are mean ± s.e.m, *p < 0.05, **p < 0.01, ***p < 0.001, and ****p < 0.0001, ns: not significant.

To identify molecular factors determining circadian variations of myocardial injury, we conducted RNA-sequencing (RNA-Seq) analysis on the area-at-risk (AAR) from C57BL/6J mice subjected to myocardial IRI at two distinct time points: ZT8 and ZT20, corresponding to the times of minimum and maximum observed cardiac injuries, respectively. Principal component analysis (PCA) (Extended Data Fig. 2a) and the hierarchical clustering of differentially expressed genes (DEGs) (Fig. 1a) both revealed distinct transcriptional signatures between the different ZT groups. We identified 18 upregulated and 42 downregulated DEGs in the ZT8 mouse AAR compared to the ZT20 group (Extended Data Fig. 2b). Notably, *Bmal1* was significantly downregulated at ZT8 (Fig. 1a.b). Furthermore, we discovered an antiphasic expression pattern for several well-known BMAL1 target genes in the circadian rhythm family, such as *Per2*, *Per3*, *Nr1d2* and *Dbp* (Fig. 1b and Supplementary Table 1). Real-time PCR analysis validated this antiphasic rhythmic oscillation of *Bmal1* and its target genes, such as *Per2*, *Nr1d1*, and *Dbp*, in ischemic mouse hearts (Fig. 1c). The antiphasic pattern observed between *Bmal1* and its target genes can be attributed to the intricate regulatory mechanisms governing circadian rhythms^14,19,20^. This indicates that BMAL1’s transcriptional activity varies throughout the day, reaching its peak around ZT8, as evidenced by the maximum expression of its target genes. Furthermore, the negative feedback loop, driven by PERs and CRYs, suppresses BMAL1’s transcriptional activity around ZT20. This intricate regulation ensures precise temporal control of the roughly 24-hour oscillation in gene expression, potentially influencing processes such as myocardial injury^13,14,31^. Subsequent analyses provided further insights into the key node in driving these variations. Gene Ontology (GO) analysis indicated a significant enrichment in the biological process terms “rhythmic process” and “circadian rhythm” (Fig. 1d and Supplementary Table 2). The Kyoto Encyclopedia of Genes and Genomes (KEGG) pathway analysis highlighted “circadian rhythm” as one of the top five significant pathways, with *Bmal1* emerging as the most significantly downregulated gene (Extended Data Fig. 2c). To further determine the key regulators governing the daily fluctuations in DEGs, we constructed a dysregulation network composed of eight transcription factors and 25 genes (Extended Data Fig. 2d). Notably, BMAL1 emerged as the central transcription factor, underscoring its pivotal role in orchestrating the diurnal fluctuations of DEGs at the transcriptional level in ischemic mouse hearts.

To investigate transcriptional pathways in the human left ventricles experiencing myocardial injury at different times, we examined samples from a prospective study of myocardial injury in humans during cardiac surgery (clinicaltrials.gov: NCT00281164). This ongoing study involved 56 patients who underwent elective aortic valve replacement surgery (with or without coronary artery bypass grafting) in the morning (samples collected between 8:00 am-12:00 pm, median time 10:32 am) and 17 patients who underwent the same procedure in the afternoon (samples collected between 3:00–9:00 pm, median time 5:15 pm). LV biopsy samples from the morning and afternoon patient cohorts were obtained after approximately 80 minutes of aortic cross-clamping. These samples were then subjected to RNA-Seq analysis (Fig. 1e). To ensure objective comparisons between patient groups, we initially verified that both cohorts had comparable patient demographics, preoperative and intraoperative characteristics (Extended Data Table 1). To visualize the broader transcriptional landscape, we performed a PCA on these DEGs, elucidating distinct transcriptional signatures across patient groups (Extended Data Fig. 2e). Intriguingly, when we analyzed sample clustering by applying Pearson’s correlation matrix with DEGs, it became evident that the timing of surgery was a primary factor in determining the patterns of clustering. This correlation was stronger than other major covariates, such as treatment status, smoking history, pulmonary disease, and renal disease (Fig. 1f and Supplementary Table 3). Subsequently, our RNA-seq analysis identified 257 genes exhibiting differential expression based on surgical timing: specifically, 208 genes were upregulated, and 49 were downregulated in the morning surgery patients compared to their afternoon counterparts (Extended Data Fig. 2f and Supplementary Table 4). Moreover, in alignment with findings from murine myocardial injury models, *BMAL1* was identified as one of the most significantly downregulated genes in the left ventricles of morning surgery patients compared to those from the afternoon (Fig. 1g and Supplementary Table 4). GO analysis pinpointed a significant enrichment in the “circadian rhythm” and “circadian regulation of gene expression” processes (Fig. 1h and Supplementary Table 5), indicating their potential roles in the observed diurnal variation of myocardial injury. The KEGG pathway analysis further supported this, ranking the “circadian rhythm” pathway among the top three significant pathways (Extended Data Fig. 2g). Additionally, mirroring the observations in the murine studies, *BMAL1*, along with its target genes *NRID1* and *PER2*, exhibited a notable antiphasic expression pattern (Fig. 1i). Given the striking consistency between transcriptomic findings in IRI murine models and surgical patient studies, these results suggest a potential role for BMAL1 in the transcriptional control of circadian variation of myocardial injury in both mice and humans.

## Cardiomyocyte BMAL1 regulates circadian variation of myocardial injury.

**Extended Data Fig. 3.**
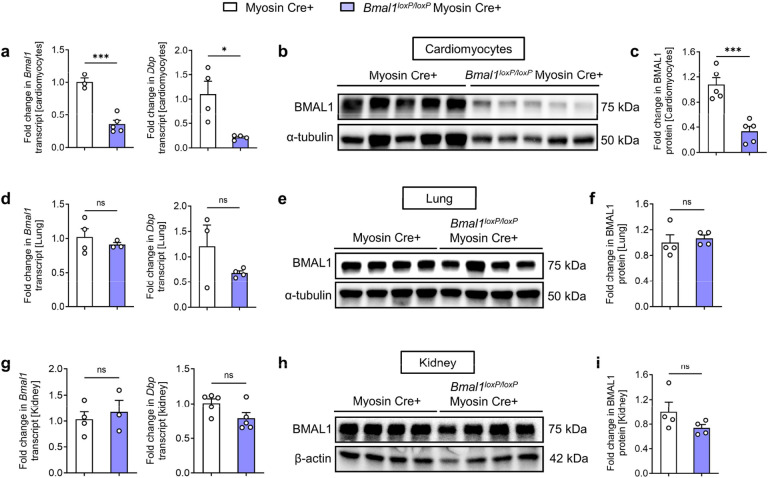
Cardiomyocyte-specific deletion of *Bmal1* in the *Bmal1*^*loxP/loxP*^Myosin Cre+ mice. *Bmal1*^*loxP/loxP*^ Myosin Cre+ mice and Myosin Cre+ mice were administered tamoxifen at 8–12 weeks of age, and the transcript levels of *Bmal1* and its target gene *Dbp* were measured one week later by real-time PCR analysis in isolated cardiomyocytes **(a)**, lungs **(b)**, and kidneys **(g)**. n = 3–4 for cardiomyocytes/Myosin Cre+ mice, n = 4–5 for cardiomyocytes/*Bmal1*^*loxP/loxP*^ Myosin Cre+ mice, n = 3–4 for lungs/Myosin Cre+ mice, n = 3–4 for lungs/*Bmal1*^*loxP/loxP*^ Myosin Cre+ mice, n = 4–5 for kidneys/Myosin Cre+ mice, n = 3–5 for kidneys/*Bmal1*^*loxP/loxP*^ Myosin Cre+ mice. BMAL1 protein levels were measured by Western blot analysis in isolated cardiomyocytes **(b)**, lungs **(e)**, and kidneys **(h)**. Quantification of BMAL1 protein levels in cardiomyocytes **(c)**, lungs **(f)**, and kidneys **(i)**. n = 5 for cardiomyocytes/Myosin Cre+ mice, n = 5 for cardiomyocytes/*Bmal1*^*loxP/loxP*^ Myosin Cre+ mice, n = 4 for lungs/Myosin Cre+ mice, n = 4 for lungs/*Bmal1*^*loxP/loxP*^ Myosin Cre+ mice, n = 4 for kidneys/Myosin Cre+ mice, n = 4 for kidneys/*Bmal1*^*loxP/loxP*^ Myosin Cre+ mice. Statistical analysis varied depending on the context: Welch’s *t* test was used for assessing *Dbp* transcript levels in cardiomyocytes and lungs, the Mann-Whitney test was employed for evaluating *Bmal1* transcript levels in the lungs, and the Student’s *t* test was applied in the analysis of the remaining figures. All data are mean ± s.e.m, *p < 0.05, and ***p < 0.001, ns: not significant.

**Extended Data Fig. 4.**
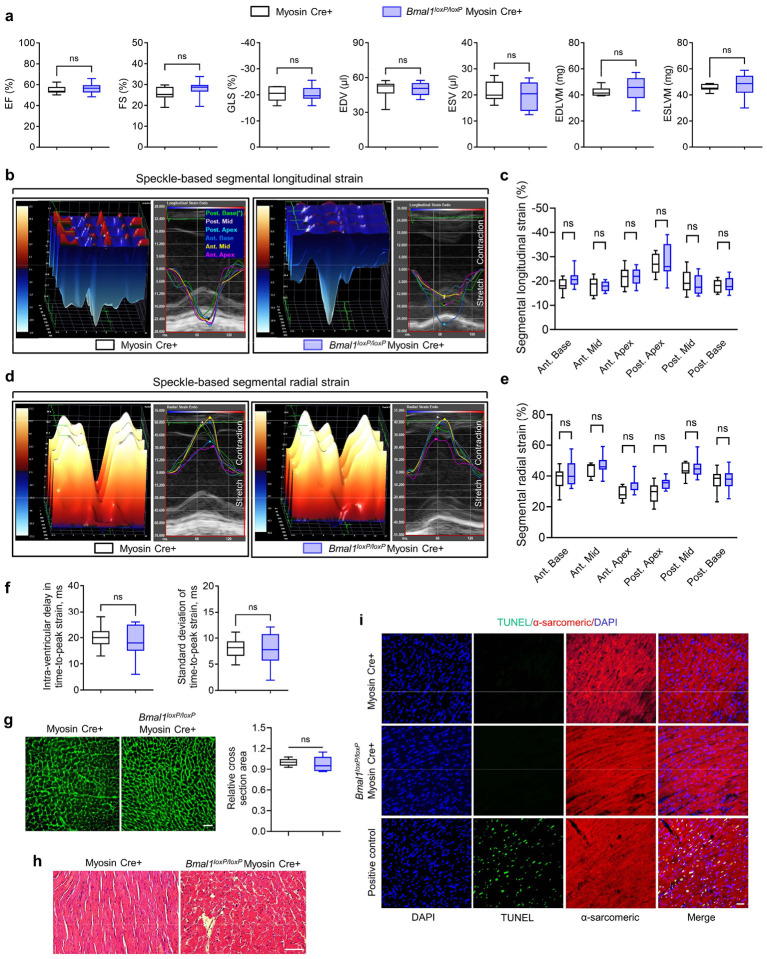
Baseline cardiac structure and function are unaffected by induced cardiomyocyte-specific *Bmal1* deletion. **a-f,** *Bmal1*^*loxP/loxP*^ Myosin Cre+ mice and Myosin Cre+ mice were administered tamoxifen at 8–12 weeks of age, and cardiac function was measured by speckle-tracking echocardiography analysis one week later. **a,** LV systolic function (EF, FS, and GLS), LV volume (EDV and ESV) and mass (EDLVM and ESLVM). Statistical analysis was performed using unpaired Student’s *t*-tests. **b,** Representative left ventricular 3D longitudinal strain (48 points) and 6-segment longitudinal strain images demonstrating wall motion patterns (Color-coded six segments: Ant. Base, Anterior Base; Ant. Mid, Anterior Middle; Ant. Apex, Anterior Apex; Post. Apex, Posterior Apex; Post. Mid, Posterior Middle; Post. Base, Posterior Base). **c,** Left ventricular segmental wall contractility detected by peak longitudinal strain. Statistical analysis was performed using two-way ANOVA. **d,** Representative left ventricular 3D long-axis radial strain (48 points) and 6-segment radial strain images demonstrating wall motion patterns. **e,** Left ventricular segmental wall contractility detected by peak radial strain. Statistical analysis was performed using unpaired Student’s *t*-tests. **f,** Left ventricular mechanical dyssynchrony as measured by intra-ventricular delay in time-to-peak strain and standard deviation of time-to-peak strain based on peak longitudinal strain. n = 9/Myosin Cre+ mice and n = 11/*Bmal1*^*loxP/loxP*^ Myosin Cre+ mice. Statistical analysis was performed using unpaired Student’s *t*-tests. **g,** Representative images of wheat germ agglutinin (WGA) showing cardiomyocyte size and quantification of myocyte cross-sectional area in mice; scale bar, 20 μm. Each quantification value dot represents the average value of three fields in one section, n = 5. Statistical analysis was performed using unpaired Student’s *t*-tests. **h,** Representative images of HE staining; scale bar, 20 μm. n = 5. **i,** Representative images of terminal deoxynucleotidyl transferase dUTP nick end labeling (TUNEL). Sections were co-stained with cardiomyocyte marker a-sarcomeric and DAPI. Incubation of sections with DNase I served as the positive control. Scale bar. 20 μm. n = 5. All data are presented as box-plots. ns: not significant.

BMAL1 is fundamental in maintaining the intrinsic biological clocks that guide daily rhythms in physiology and behavior in a wide range of organisms^14,17,19,20,50^. While earlier research has underscored the diverse functions of BMAL1 in cardiovascular health and diseases^51–56^, its mechanism on daily fluctuations of myocardial injury is yet to be examined. Consequently, we delved into the functional role of BMAL1 in cardiomyocytes to explore its contribution to the circadian modulation of cardiac injury, given the profound implications of cardiomyocyte dysfunction and death in MI progression^57,58^. Previous research has shown that cardiomyocyte-specific *Bmal1* knockout can lead to age-dependent hypertrophic cardiomyopathy and impaired cardiac performance in mice between 24 and 36 weeks of age^51,56,59^. To circumvent cardiomyopathy associated with prolonged *Bmal1* deletion, we generated an inducible cardiomyocyte-specific *Bmal1* knockout mouse line (*Bmal1*^*loxP/loxP*^ Myosin Cre+ mice) by crossing *Bmal1* floxed (*Bmal1*^*loxP/loxP*^) mice with Myosin Cre+ mice. At 8 weeks of age, we induced *Bmal1* gene ablation via intraperitoneal (i.p.) tamoxifen injection. Both real-time PCR and Western blot analyses confirmed the efficient depletion of *Bmal1*, specifically in cardiomyocytes, but not in the lungs or kidneys (Extended Data Fig. 3). Consistent with previous studies^60^, short-term *Bmal1* deletion in cardiomyocytes showed no signs of cardiac dysfunction or variations in cardiomyocyte size, structure, or apoptosis compared with control mice at baseline (Extended Data Fig. 4).

**Extended Data Fig. 5.**
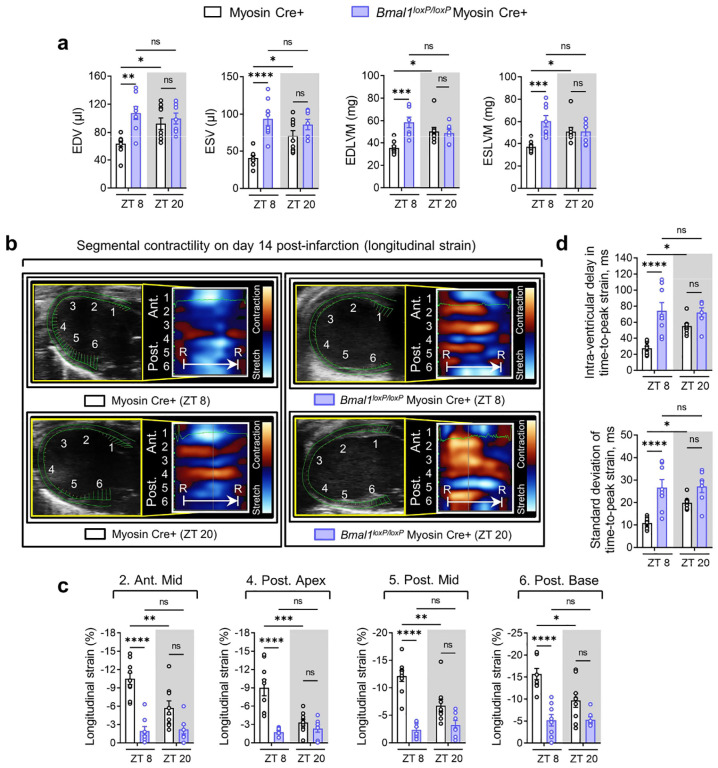
Cardiomyocyte-specific *Bmal1* deletion diminishes the circadian variation of cardiac function impairment. Cardiac function was evaluated by speckle-tracking echocardiography analysis on day 14 post-MI in *Bmal1*^*loxP/loxP*^ Myosin Cre+ mice and Myosin Cre+ mice subjected to IRI at ZT8 or ZT20. **a,** LV volume (EDV and ESV) and mass (EDLVM and ESLVM). **b,** Representative B-mode images from the left ventricular long-axis view with 2D longitudinal strain analysis, showing 6 segments (1. Ant. Base, Anterior Base; 2. Ant. Mid, Anterior Middle; 3. Ant. Apex, Anterior Apex; 4. Post. Apex, Posterior Apex; 5. Post. Mid, Posterior Middle; 6. Post. Base, Posterior Base). **c,** Left ventricular segmental wall contractility detected by peak longitudinal strain. **d,** Left ventricular mechanical dyssynchrony as measured by intra-ventricular delay in time-to-peak strain and standard deviation of time-to-peak strain based on peak longitudinal strain. a-d: n = 7/*Bmal1*^*loxP/loxP*^ Myosin Cre+ mice/time point and n = 9 for Myosin Cre+ mice/time point. All data are mean ± s.e.m. Statistical analysis was performed using two-way ANOVA. *p < 0.05, **p < 0.01, ***p < 0.001, and ****p < 0.0001, ns: not significant,

We then subjected *Bmal1*^*loxP/loxP*^ Myosin Cre+ mice and Myosin Cre+ mice to myocardial IRI at ZT8 or ZT20 (Fig. 1j). After 2h of reperfusion, we observed that *Bmal1*^*loxP/loxP*^ Myosin Cre+ mice exhibited a loss of inherent circadian variability in myocardial injury and diminished endogenous cardioprotection at ZT8 compared to their control counterparts (Fig. 1k–n). We next investigated whether *Bmal1* deletion in cardiomyocytes influenced the circadian phenotype of cardiac injury during the extended reperfusion periods (day 14 post-MI). Similar to our findings in the acute phase, the time-of-day difference in long-term cardiac impairment was also diminished in *Bmal1*^*loxP/loxP*^ Myosin Cre+ mice. Detailed STE analysis revealed that *Bmal1*^*loxP/loxP*^ Myosin Cre+ mice subjected to IRI at ZT8 exhibited a significant decline in systolic function (ejection fraction [EF], fractional shortening [FS], global longitudinal strain [GLS]) (Fig. 1o) and showed increased LV dilation (end-diastolic volume [EDV] and end-systolic volume [ESV]) and LV mass (end-diastolic left ventricular mass [EDLVM] and end-systolic left ventricular mass [ESLVM]) compared to controls (Extended Data Fig. 5a). Notably, these mice also displayed reduced contractility in both ‘infarcted’ (anterior mid and posterior apex) and ‘non-infarcted’ (posterior mid and posterior base) heart segments, indicating more severe and broader myocardial injury (Extended Data Fig. 5b,c). Significant intra-ventricular disparities were also evident in these mice, suggesting potential progression of heart failure^46–49^ (Fig. 1p and Extended Data Fig. 5d). However, when IRI was performed at ZT20, there was no significant difference in the extent of cardiac function impairment between *Bmal1*^*loxP/loxP*^ Myosin Cre+ mice and Myosin Cre+ mice (Fig. 1o,p and Extended Data Fig. 5). Together, our findings highlight the pivotal role of cardiomyocyte BMAL1 in governing the circadian variation of myocardial injury and cardiac dysfunction in both the acute phase and extended reperfusion period.

## BMAL1 interacts with hypoxia-inducible factor HIF2A during ambient hypoxia

**Extended Data Fig. 6.**
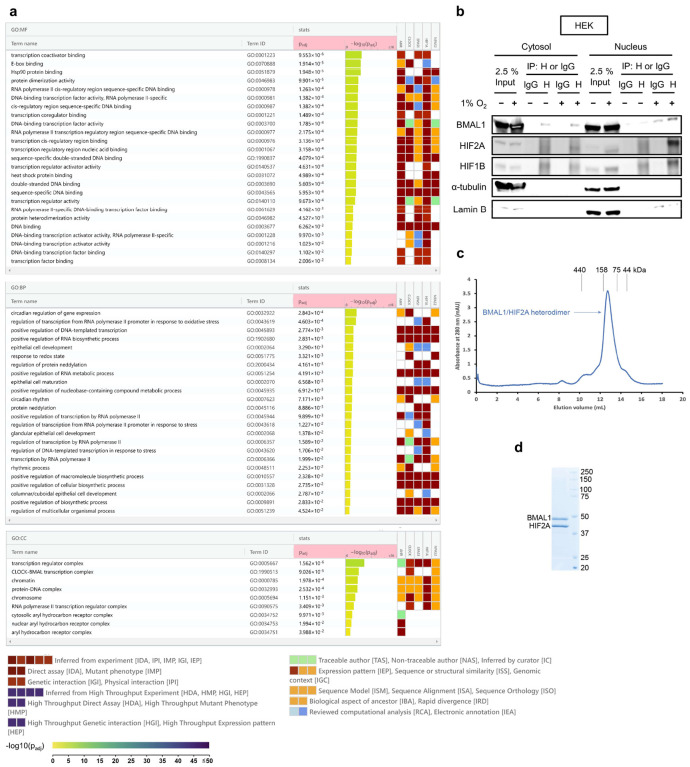
Interaction of BMAL1 and HIF2A. **a,** GO enrichment analysis was conducted to elucidate the molecular functions (MF), biological processes (BP), and cellular components (CC) associated with proteins predicted by the HuRI to potentially interact with BMAL1. **b,** Western Blot analysis of reciprocal co-IP with HIF2A in hypoxia-treated (1% O_2_, 4h) or normoxia-treated HEK293 cells. Cytosolic and nuclear protein extracts were immunoprecipitated with HIF2A and blotted with anti-BMAL1, anti-HIF2A, anti-HIF1B, anti-α-tubulin, and anti-Lamin B antibodies. An IgG control affirmed procedure specificity. H indicates HIF2A. n = 3. **c,** Size-exclusion chromatography analysis of the BMAL1/HIF2A heterodimer. The purified BMAL1/HIF2A complex was loaded onto a Superdex 200 Increase 10/300 GL column. n = 3. The molecular weights of makers are as indicated. **d,** SDS-PAGE analysis of the recombinant BMAL1/HIF2A heterodimer purified by size-exclusion chromatography. n = 3.

**Fig. 2.**
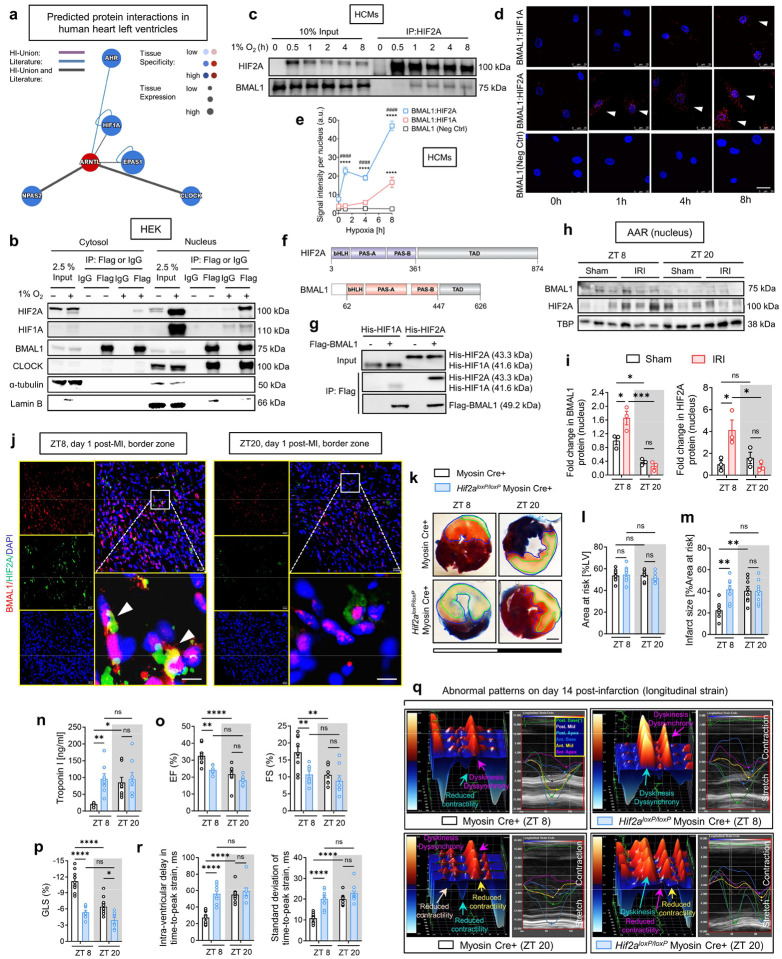
HIF2A interacts with BMAL1 and regulates circadian variation of myocardial injury. **a,** Predicted protein-protein interactions between BMAL1 and other bHLH-PAS transcription factors using the Human Reference Interactome (HuRI) in the left ventricles of human hearts. EPAS1, endothelial PAS domain protein 1 (also known as HIF2A); NPAS, neuronal PAS domain protein; AHR, aryl hydrocarbon receptor; HI-Union, a dataset specifically represents a combined set of protein-protein interactions identified through various high-throughput Yeast two-Hybrid screens performed by the HuRI project; Literature, protein-protein interactions that have been identified and reported in scientific literature. **b,** Western blot analysis of co-IP in HEK293 cells overexpressing BMAL1-Flag. Cytosolic and nuclear protein extracts from hypoxia- (1% O_2_, 4 h) or normoxia-treated HEK293 cells were immunoprecipitated with Flag and blotted with anti-HIF2A, anti-HIF1A, anti-BMAL1, anti-CLOCK, anti-α-tubulin, and anti-Lamin B antibodies. An IgG control affirmed procedure specificity. n = 3 independent experiments. **c,** Western blot analysis of co-IP with HIF2A in HCMs following 1% O_2_ treatment for indicated times (0h, 0.5h, 1h, 2h, 4h, and 8h). n = 3 independent experiments. **d** and **e,** HCMs were treated with 1% O_2_ for indicated times (0h, 1h, 4h, and 8h), and PLA was performed between BMAL1 and HIF2A, as well as BMAL1 and HIF1A. Representative confocal images **(d)** and corresponding quantification of PLA signals per nucleus **(e)** are presented; Scale bar, 25 μm. White arrows indicate the close interaction between BMAL1:HIF2A or BMAL1:HIF1A in the nuclei. n = 20/BMAL1:HIF2A, n = 20/BMAL1:HIF1A, and n = 10/BMAL1 negative control, independent experiments. Statistical analysis was performed using two-way ANOVA. ####p < 0.0001 compared to BMAL1:HIF1A; ****p < 0.0001 compared to BMAL1 negative control. **f,** Schematic illustration of the HIF2A (purple) and BMAL1 (orange) proteins with functional domains indicated. TAD, transactivation domain. **g,** Flag pull-down analysis. Recombinant Flag-tagged BMAL1 immobilized on anti-Flag M2 beads was used to pull down purified His-tagged HIF2A or His-tagged HIF1A. The elutes were analyzed by Western blot analysis. n = 3 independent experiments. **h** and **i**, **h,** C57BL/6J mice were subjected to myocardial IRI at ZT8 or ZT20, and protein levels of BMAL1 and HIF2A in the nuclear extracts from the AAR following 2h of reperfusion were measured by Western blot analysis. **i,** Quantification of the protein levels in **(h)**. n = 3 mice/group/time point. Statistical analysis was performed using two-way ANOVA. **j,** Representative images of fluorescence immunostaining for BMAL1 (red) and HIF2A (green) in the border zone of the hearts of C57BL/6J mice on day 1 post-MI; Scale bar, 50 μm, white arrows indicate the colocalization of BMAL1 and HIF2A in the nuclei. n = 3 mice/group/time point. **k,** Representative heart slices subjected to Evan’s blue and TTC double staining after 2 h of reperfusion: infarct area (green line) and AAR (blue line); scale bar, 1 mm. **l,** Percentage of the AAR relative to the size of the LV. n = 7 mice/group/time point. Statistical analysis was performed using two-way ANOVA. **m,** Infarct sizes represented as the percentage of the AAR. n = same as above. Statistical analysis was performed using two-way ANOVA. **n,** Serum troponin I levels were evaluated at 2 hours after reperfusion. n = 8 mice/group/time point. Statistical analysis was performed using two-way ANOVA. **o**-**r**, Cardiac function was evaluated by speckle-tracking echocardiography on day 14 post-MI. LV systolic function, including EF, FS **(o)** and GLS **(p)**, was measured. **q,** Representative left ventricular 3D (48 points) longitudinal strain and 6-segment longitudinal strain images demonstrating wall motion abnormalities (Color-coded six segments: Ant. Base, Anterior Base; Ant. Mid, Anterior Middle; Ant. Apex, Anterior Apex; Post. Apex, Posterior Apex; Post. Mid, Posterior Middle; Post. Base, Posterior Base). **r,** Left ventricular mechanical dyssynchrony as measured by intra-ventricular delay in time-to-peak strain and standard deviation of time-to-peak strain based on peak longitudinal strain. n = 8/*Hif2a*^*loxP/loxP*^ Myosin Cre+ mice/time point and n = 9/Myosin Cre+ mice/time point. Statistical analysis was performed using two-way ANOVA. All data are mean ± s.e.m, *p < 0.05, **p < 0.01, ***p < 0.001, and ****p < 0.0001, ns: not significant.

Previous studies have shown that the transcriptional activity of BMAL1 exhibits a circadian rhythm, peaking around ZT8^14,19,20,50^, which coincides with the time window of least myocardial injury (Extended Data Figs. 1 and 2h). This alignment strongly suggests that BMAL1’s transcriptional activity and its downstream targets play a crucial role in providing endogenous cardioprotection during this critical period. We next explored the mechanisms underlying this circadian-dependent cardioprotection. As a key member of the bHLH-PAS family, BMAL1 interacts with other transcription factors within this family (e.g., CLOCK) and forms transcriptionally active heterodimers to regulate circadian rhythms and various physiological processes^14,19,20,50^. In line with this, our initial step was to identify potential BMAL1 partners that might be instrumental in modulating the diurnal variations in myocardial injury. To achieve this, we utilized the Human Reference Interactome (HuRI)^61^ to predict potential interactions between BMAL1 and other bHLH-PAS transcription factors within human left ventricles. Notably, among these potential BMAL1 interactors, the endothelial PAS domain protein 1 (EPAS1), also known as HIF2A^2,4,6,23,62^ (referred to as HIF2A thereafter), emerged as the most abundantly expressed in human hearts (represented as the largest node in Fig. 2a). Subsequent GO enrichment analysis further revealed the potential interactive roles of HIF2A and BMAL1, with both proteins being highly enriched in several shared pathways (Extended Data Fig. 6a and Supplementary Table 6). These pathways encompass molecular functions such as transcription coactivator binding, protein dimerization, and DNA-binding transcription factor activity, as well as biological processes including the regulation of transcription from RNA polymerase II promoter in response to oxidative stress and the regulation of DNA-templated transcription under stress, indicating a significant role for both HIF2A and BMAL1 in cellular responses to stress and transcriptional regulation. Given the severe hypoxia experienced by cardiac tissues during MI^2,4,6,23,63^ and the pivotal role of HIF2A in maintaining oxygen homeostasis^63–66^, our research then focused on exploring the interactions between BMAL1 and HIF2A under hypoxic conditions. We first conducted co-immunoprecipitation (co-IP) and reciprocal co-IP assays using cytosol and nuclear fractions from HEK293 cells overexpressing BMAL1-Flag. We found that BMAL1 predominantly interacted with HIF2A in the cell nucleus during ambient hypoxia (1% oxygen, 4h; Fig. 2b and Extended Data Fig. 6b). This interaction was distinct from the canonical BMAL1-CLOCK interaction, which remained unaffected by changes in oxygen levels (Fig. 2b). Interestingly, despite the significant sequence similarity between HIF1A and HIF2A^23^, our experiments revealed a considerably weaker association between BMAL1 and HIF1A (Fig. 2b). This finding is consistent with previous reports showing an inefficient heterodimer formation between HIF1A and BMAL1 in yeast two-hybrid systems^67^. We further validated our findings in primary human cardiomyocytes (HCMs), demonstrating the interaction between endogenous BMAL1 and hypoxia-stabilized HIF2A (Fig. 2c). Additionally, an in-situ proximity ligation assay (PLA) conducted on HCMs demonstrated a substantial interaction between BMAL1 and HIF2A within the cellular nucleus after 1% oxygen treatment for indicated times (1h, 4h, and 8h) (Fig. 2d, e). In contrast, minimal interaction was observed between BMAL1 and HIF1A, emphasizing the specificity of the BMAL1/HIF2A interaction in human cardiomyocytes during ambient hypoxia (Fig. 2d, e).

Next, we proceeded to determine whether this interaction is direct. Both the N-terminal regions of BMAL1 and HIF2A contain a conserved bHLH DNA binding domain and two tandemly positioned PAS-A and PAS-B domains, which are responsible for dimerization with their partners^15,62^ (Fig. 2f). Their C-terminal regions include an intrinsically disordered transactivation domain (TAD), enabling their participation in transcriptional activation^62^. Pull-down analysis using purified recombinant N-terminal regions of Flag-tagged BMAL1 and His-tagged HIF2A demonstrated an apparent binding between these regions (Fig. 2g). Our result was further supported by size-exclusion chromatography analysis (Extended Data Fig. 6c, d), revealing that BMAL1 and HIF2A can form a stable heterodimer in stoichiometry, providing strong evidence of their direct physical association. Notably, we also did not observe significant binding between BMAL1 and HIF1A (Fig. 2g), even though HIF1A and HIF2A share 67% of sequence identity between their N-terminal portions. Together, our results demonstrate that the interaction between BMAL1 and HIF2A is specific and primarily mediated by their N-terminal regions.

## HIF2A co-localizes with BMAL1 in the nucleus after IRI and regulates circadian variation of myocardial injury

**Extended Data Fig. 7.**
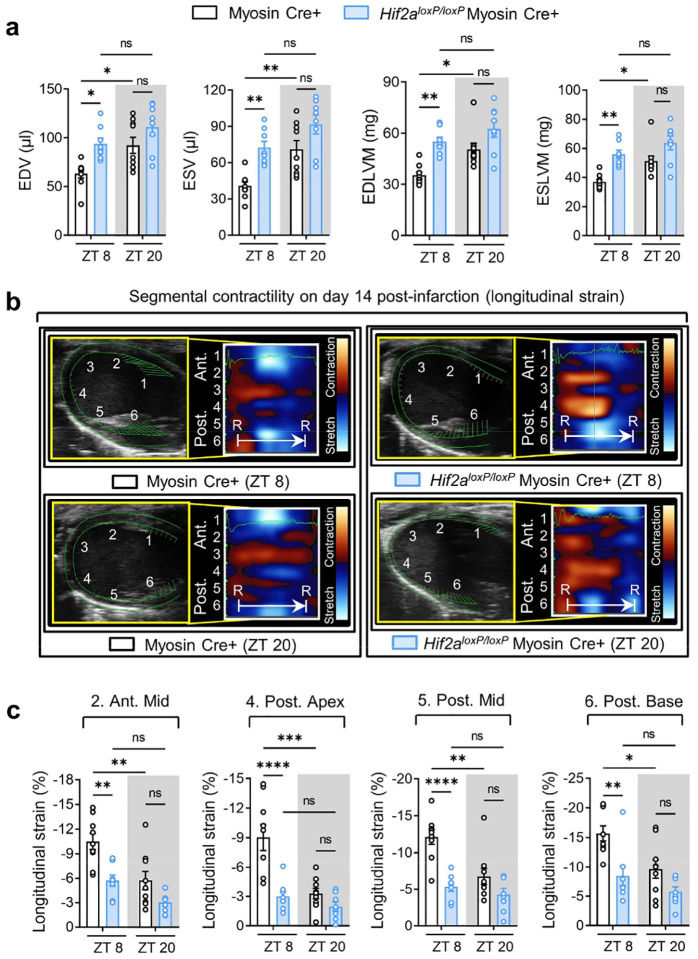
Cardiomyocyte-specific *Hif2a* deletion diminishes the circadian variation of cardiac function impairment. Cardiac function was evaluated by speckle-tracking echocardiography analysis on day 14 post-MI in *Hif2a*^*loxP/loxP*^ Myosin Cre+ mice and Myosin Cre+ mice subjected to IRI at ZT8 or ZT20. **a,** Left ventricular volume (EDV and ESV) and mass (EDLVM and ESLVM). **b,** Representative B-mode images from the left ventricular long-axis view with 2D longitudinal strain analysis, showing 6 segments (1. Ant. Base, Anterior Base; 2. Ant. Mid, Anterior Middle; 3. Ant. Apex, Anterior Apex; 4. Post. Apex, Posterior Apex; 5. Post. Mid, Posterior Middle; 6. Post. Base, Posterior Base). **c,** Left ventricular segmental wall contractility detected by peak longitudinal strain. a-c: n = 8/*Hif2a*^*loxP/loxP*^ Myosin Cre+ mice/time point, n = 9/Myosin Cre+ mice/time point. All data are mean ± s.e.m. Statistical analysis was all performed using two-way ANOVA. *p < 0.05, **p < 0.01, ***p < 0.001, and ****p < 0.0001, ns: not significant,

**Extended Data Fig. 8.**
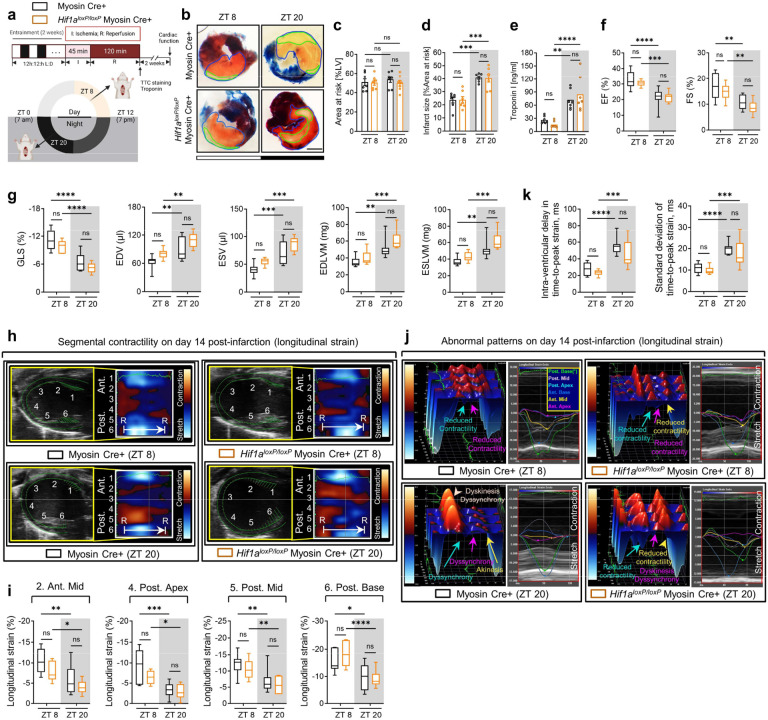
Cardiomyocyte-specific *Hif1a* deletion does not influence circadian-dependent myocardial injury. **a,** Experimental setup for evaluation of myocardial injury and cardiac function in *Hif1a*^*loxP/loxP*^ Myosin Cre+ mice and Myosin Cre+ mice subjected to IRI at ZT8 or ZT20. **b-e, b,** Representative heart slices subjected to Evan’s blue and TTC double staining following 2h of reperfusion: infarct area (green line) and AAR (blue line); scale bar, 1 mm. **c,** Percentage of the AAR relative to the size of the LV. **d,** Infarct sizes represented as the percentage of the AAR. **e,** Serum troponin I levels. b-e: n = 7 mice/group/time point. Data are mean ± s.e.m. Statistical analysis was performed using two-way ANOVA. **f-k,** Cardiac function was evaluated by speckle-tracking echocardiography analysis on day 14 post-MI. **f,** EF and FS. **g,** GLS, EDV, ESV, EDLVM, and ESLVM. **h,** Representative B-mode images from the left ventricular long-axis view with 2D longitudinal strain analysis, showing 6 segments (1. Ant. Base, Anterior Base; 2. Ant. Mid, Anterior Middle; 3. Ant. Apex, Anterior Apex; 4. Post. Apex, Posterior Apex; 5. Post. Mid, Posterior Middle; 6. Post. Base, Posterior Base). **i,** Left ventricular segmental wall contractility detected by peak longitudinal strain. **j,** Representative left ventricular 3D (48 points) longitudinal strain and 6-segment longitudinal strain images demonstrating wall motion abnormalities. **k,** Left ventricular mechanical dyssynchrony as measured by intra-ventricular delay in time-to-peak strain and standard deviation of time-to-peak strain based on peak longitudinal strain. f-k: n = 9 mice/group/time point. Data are presented as box-plots. Statistical analysis was performed using two-way ANOVA. *p < 0.05, **p < 0.01, ***p < 0.001, and ****p < 0.0001, ns: not significant.

While prior research has highlighted HIF2A’s cardioprotective effects against myocardial IRI^63,66,68^, its potential influence on diurnal variations of myocardial injury is presently unknown. Building upon our discovery of a direct interaction between BMAL1 and HIF2A, we proceeded to examine the influence of HIF2A on circadian-dependent cardiac injury. Initial observations revealed that after 2h of reperfusion, there was a notable stabilization of HIF2A protein in the nuclear fraction of the mouse AAR at ZT8 compared to ZT20 (Fig. 2h, i). Intriguingly, this pattern mirrored that of BMAL1 protein levels after IRI (Fig. 2h, i). Additionally, on day 1 post-MI, the immunofluorescence staining demonstrated a significant co-localization of BMAL1 and HIF2A within the nuclei of cells in the border zones (areas of ischemic heart tissue) (Fig. 2j). This co-localization was particularly evident in mice exposed to IRI at ZT8, underscoring a circadian-dependent expression and interaction between BMAL1 and HIF2A in the ischemic regions of mouse hearts (Fig. 2j). To further elucidate the functional role of HIF2A in circadian variation of myocardial injury, we adopted a previously described mouse model with induced deletion of myocyte-specific *Hif2a* (*Hif2a*^*loxP/loxP*^ Myosin Cre+)^66,68^. These mice were then subjected to myocardial IRI at either ZT8 or ZT20. Similar to our findings in *Bmal1*^*loxP/loxP*^ Myosin Cre+ mice, *Hif2a*^*loxP/loxP*^ Myosin Cre+ mice exhibited abolished circadian-dependent myocardial injury and diminished endogenous cardioprotection at ZT8, evident from increased infarct sizes and elevated serum troponin I levels following 2h of reperfusion (Fig. 2k–n). Furthermore, in support of our findings in the acute phase, the diurnal variability in cardiac function impairment during the extended reperfusion period was also dampened in *Hif2a*^*loxP/loxP*^ Myosin Cre+ mice. STE analysis conducted on day 14 post-MI revealed that *Hif2a*^*loxP/loxP*^ Myosin Cre+ mice subjected to IRI at ZT8 exhibited a significant decline in cardiac systolic function, as evidenced by reduced EF, FS, and GLS (Fig. 2o, p), along with more pronounced LV dilation and increased LV mass (Extended Data Fig. 7a). Additionally, exacerbated wall motion abnormalities and intra-ventricular disparities were distinctly seen in *Hif2a*^*loxP/loxP*^ Myosin Cre+ mouse exposed to IRI at ZT8, indicating severe myocardial injury and progression toward heart failure (Fig. 2q, r and Extended Data Fig. 7b, c). However, when IRI was performed at ZT20, there was no significant difference in cardiac injury between *Hif2a*^*loxP/loxP*^ Myosin Cre+ mice and Myosin Cre+ mice by day 14 post-MI (Fig. 2q, r and Extended Data Fig. 7). Given that previous studies have implicated HIF1A in cardioprotection^5,24^, we subsequently examined the impact of cardiomyocyte HIF1A on the circadian dependence of cardiac injury. Contrary to our findings with HIF2A, the induced deletion of *Hif1a* in cardiomyocytes did not influence the circadian variation of myocardial injury in both the acute and the extended reperfusion phases (Extended Data Fig. 8). These findings unravel a distinct role for cardiomyocyte HIF2A in modulating the diurnal susceptibility of the myocardium to IRI.

## Circadian regulation of AREG by BMAL1/HIF2A heterodimer

**Fig. 3.**
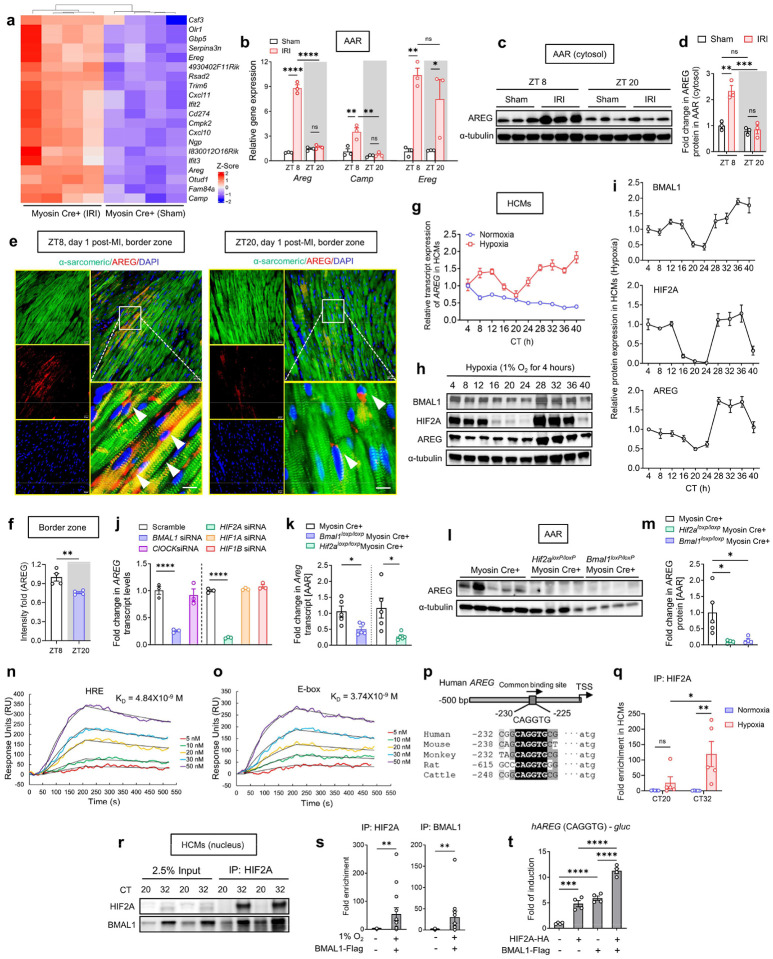
*Areg* is a circadian-dependent target of the BMAL1/HIF2A heterodimer. **a,** Heatmap with the expression patterns of the top 20 potential HIF2A target genes (reanalyzed our previously published microarray data^66^). n = 4 mice/group. **b,** Potential HIF2A target genes upregulated at ZT8 in the AAR from C57BL/6J mice following 2h of reperfusion. n = 3 mice/group/time point. Statistical analysis was performed using two-way ANOVA. **c,** AREG protein levels in the cytosolic extracts were measured by Western blot analysis. n = 3 mice/group/time point. **d,** Quantification of the AREG protein levels in **(c).** n = same as above. Statistical analysis was performed using two-way ANOVA. **e,** Representative immunostaining of AREG (red), α-sarcomeric (cardiomyocyte marker; green), and nuclei (DAPI; blue) on day 1 post-MI in the border zone of C57BL/6J mouse hearts subjected to myocardial IRI at ZT8 or ZT20; Scale bar, 50 μm. Arrows indicate α-sarcomeric^+^/AREG^+^ cells. n = 4 mice/time point. **f,** Quantification of AREG fluorescence intensity in **(e).** n = same as above. Statistical analysis was performed using unpaired Student’s *t*-tests. **g**-**i,** HCMs were synchronized by dexamethasone (200 nM, 1h) and then exposed to normoxia or hypoxia for 4h (1% O_2_) at different intervals post-synchronization (CT4 to CT40). **g,** *AREG* transcript levels were measured by real-time PCR analysis. **h,** Protein levels of BMAL1, HIF2A or AREG were measured by Western blot analysis. **i,** Quantification of protein levels in **(h).** n = 3 independent experiments. **j,** HEK293 cells were transfected with *BMAL1* siRNA, *CLOCK* siRNA, *HIF2A* siRNA, *HIF1A* siRNA, *HIF1B* siRNA (50 nM), or Scrambled siRNA for six hours and exposed to hypoxia for 4h (1% O_2_). *AREG* transcript levels were then measured by real-time PCR analysis. n = 3 independent experiments. Statistical analysis was performed using one-way ANOVA. **k,** Transcript levels of *AREG* were evaluated by real-time PCR analysis following 2h of reperfusion in the AAR of hearts from Myosin Cre+ mice, *Hif2a*^*loxP/loxP*^ Myosin Cre+ mice and *Bmal1*^*loxP/loxP*^ Myosin Cre+ mice. n = 5 mice independent experiments. Statistical analysis was performed using unpaired Student’s *t*-tests. **l,** AREG protein levels were evaluated by Western blot analysis following 2h of reperfusion in the AAR of hearts from *Bmal1*^*loxP/loxP*^ Myosin Cre+ mice, *Hif2a*^*loxP/loxP*^ Myosin Cre+ mice and Myosin Cre+ mice. n = 5/Myosin Cre+, n = 4/*Hif2a*^*loxP/loxP*^ Myosin Cre+ mice, and n = 4/*Bmal1*^*loxP/loxP*^ Myosin Cre+ mice independent experiments. **m,** Quantification of AREG protein levels in **(l)**. n = same as above. Statistical analysis was performed using one-way ANOVA. **n** and **o**, SPR analysis of the interaction between BMAL1/HIF2A and DNA. Biotinylated HRE DNA **(n)** or E-box DNA **(o)** was immobilized on a sensor. Protein concentrations of the BMAL1/HIF2A heterodimer used for measurements are indicated. n = 3 independent experiments. **p,** A highly conserved putative common binding site (CAGGTG) for both BMAL1 and HIF2A on the *AREG* promoter was predicted by JASPAR^70^ (https://jaspar.elixir.no/). **q,** HCMs were synchronized by dexamethasone (200 nM, 1h) and exposed to normoxia or hypoxia for 4h (1% O_2_) at CT20 or CT32. ChIP-qPCR assays were conducted using HIF2A to examine the binding of hypoxia-induced HIF2A to the common binding site on the human *AREG* promoter. IgG was used as a negative control. n = 5 independent experiments. Statistical analysis was performed using two-way ANOVA. **r,** Synchronized HCMs after hypoxia treatment (1% O_2_, 4h) were collected at CT20 or CT32, nuclear protein extracts were immunoprecipitated with HIF2A and blotted with anti-HIF2A and anti-BMAL1 antibodies. n = 3 independent experiments. **s,** HEK293 cells were transfected with BMAL1-Flag and exposed to normoxia or hypoxia (1% O_2_) for 4h. ChIP-qPCR assays were conducted by HIF2A or BMAL1 antibodies to examine their binding to the human *AREG* promoter. n = 10–13 independent experiments. Statistical analysis was performed using two-way ANOVA. Outliers have been identified and removed using the ROUT (Q = 1%) method in GraphPad Prism. **t,** HEK293 cells were transfected with HIF2A-HA, BMAL1-Flag, or both, and luciferase assays were conducted to examine the transcription activation activity of the BMAL1/HIF2A complex on human *AREG* promoter bearing the common binding site. n = 4 independent experiments. Statistical analysis was performed using one-way ANOVA. All data are mean ± s.e.m. All *t*-tests were two-tailed. *p < 0.05, **p < 0.01, ***p < 0.001, and ****p < 0.0001, ns: not significant.

Next, we aimed to investigate the mechanisms by which the BMAL1/HIF2A influences the diurnal variations of myocardial injury. By revisiting our previously published microarray data^66^, we compared transcript levels in post-ischemic myocardial tissue from Myosin Cre+ and *Hif2a*^*loxP/loxP*^ Myosin Cre+ mice. This analysis unveiled 43 potential HIF2A target genes that were uniquely upregulated in Myosin Cre+ mice following IRI (Supplementary Table 7). We subsequently focused on the expression patterns of the top 20 potential HIF2A target genes (Fig. 3a). Building upon our discovery that cardiomyocyte HIF2A stabilization peaks around ZT8 after IRI, and its deletion diminishes endogenous cardioprotection at this time point, we examined whether these top 20 putative HIF2A target genes also follow a circadian pattern post-MI. Interestingly, we observed a significant upregulation of several potential HIF2A target genes at ZT8, including *Areg*, *Camp*, and *Ereg* (Fig. 3b). Notably, the *Areg* gene, a member of the epidermal growth factor family and a known HIF2A target gene^69^, demonstrated the most pronounced increase, with over a 5-fold rise in the AAR in the ZT8 group compared to the ZT20 group (Fig. 3b and Extended Data Table 2). AREG protein levels in cytosolic extracts also showed a similar diurnal pattern (Fig. 3c, d). Importantly, this fluctuation paralleled the expression pattern of the BMAL1/HIF2A complex in the nucleus, peaking at ZT8 (Fig. 2h–j). Furthermore, the peak induction of *Areg* at ZT8 corresponds with both the zenith of BMAL1’s transcriptional activity and the least myocardial injury, suggesting a potential role of AREG in circadian-dependent cardioprotection driven by the BMAL1/HIF2A complex at this time.

**Extended Data Fig. 9.**
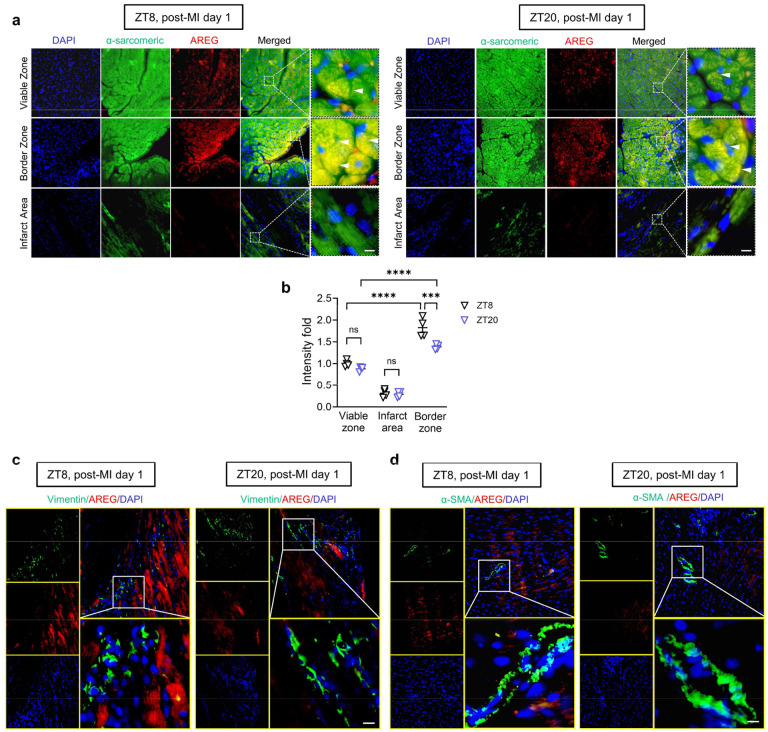
Increased induction of AREG in cardiomyocytes in the border zone from mouse hearts on day 1 post-MI. **a-b, a,** Representative immunostaining of AREG (red), α-sarcomeric (cardiomyocyte marker; green), and nuclei (DAPI; blue) on day 1 post-MI in the border zone, infarct area, and viable zone of hearts from C57BL/6J mice subjected to myocardial IRI at ZT8 or ZT20, scale bar, 50 μm. Arrows indicate α-sarcomeric^+^/AREG^+^ cells. **b,** Quantification of fluorescence intensity of AREG in **(a)**. Normalized to the AREG levels in the viable zone at ZT8. Each quantification value dot represents the average value of three fields in one section. n = 4 mice/group/time point. Data are mean ± s.e.m. Statistical analysis was performed using two-way ANOVA. ***p < 0.001, and ****p < 0.0001, ns: not significant. **c-d,** Representative immunostaining of AREG (red), vimentin (fibroblast marker; green) **(c)**, α-smooth muscle actin (α-SMA, smooth muscle cell marker; green) **(d)** and nuclei (DAPI; blue) on day 1 post-MI in the border zone of hearts from C57BL/6J mice subjected to myocardial IRI at ZT8 or ZT20, scale bar, 50 μm. n = 3–5 mice/group/time point.

To further elucidate the spatiotemporal expression of AREG following IRI, we examined the AREG expression in distinct regions: the ischemic areas (infarct area and border zone) and the unaffected myocardium (viable zone) of mice subjected to IRI at ZT8 or ZT20. We observed that AREG levels remained elevated in the cytoplasm of cardiomyocytes in the border zones on day 1 post-MI, with notably higher induction at ZT8 compared to ZT20 (Fig. 3e, f and Extended Data Fig. 9a, b). Conversely, both the infarct areas and viable zones displayed significantly lower AREG levels (Extended Data Fig. 9a, b). To explore whether fibroblasts or smooth muscle cells also contributed to the increased AREG expression after MI, we conducted double-immunofluorescence staining for AREG along with fibroblast marker (Vimentin) and smooth muscle cell marker (α-smooth muscle actin, α-SMA). The results showed that AREG expression was very low in these cells in ischemic mouse hearts following IRI (Extended Data Fig. 9c, d). This spatially localized and time-dependent induction of AREG in cardiomyocytes suggests a dual-regulatory mechanism at play: the persistent effects of hypoxia within the border zone intertwined with circadian rhythms. This notion is further supported by the increased co-localization of BMAL1 and HIF2A in the cell nucleus in the border zone at ZT8 relative to ZT20 (Fig. 2j). These findings imply that the BMAL1/HIF2A complex may play a pivotal role in orchestrating the diurnal induction of AREG in mouse hearts following IRI.

To delineate the impact of the cell-intrinsic molecular clock on *AREG* induction in cardiomyocytes, separate from external systemic cues originating from the suprachiasmatic nucleus and associated neuronal and humoral signals^14,19,20^, we employed an in vitro model. Initially, HCMs were synchronized by dexamethasone and then exposed to ambient hypoxia for 4h (1% oxygen) at different intervals post-synchronization (CT4 to CT40). *AREG* transcript levels displayed no rhythmicity under normoxia (Fig. 3g). Intriguingly, after hypoxia treatment, AREG expression at both the mRNA and protein levels was notably induced at CT32–36, with a much weaker induction at CT20–24 (Fig. 3g–3i). This mirrored the diurnal expression pattern of the BMAL1/HIF2A complex in HCMs (Fig. 3h, i), suggesting a hypoxia-driven regulatory mechanism involving BMAL1 and HIF2A in *AREG* induction.

We then investigated whether the induction of *AREG* depends on the BMAL1/HIF2A complex. Using small interfering RNA (siRNA), we silenced *HIF2A* or *BMAL1* in HEK293 cells, which resulted in a substantial reduction in hypoxia-induced *AREG* expression (Fig. 3j). Intriguingly, *AREG* transcript levels remained unaffected by *CLOCK, HIF1A*, or *HIF1B* knockdown, indicating a specific role of HIF2A and BMAL1 in the hypoxia-driven *AREG* induction. Consistently, myocardial IRI-triggered *AREG* induction at both the transcript and protein levels was significantly compromised in the AAR from *Bmal1*^*loxP/loxP*^ Myosin Cre+ mice or *Hif2a*^*loxP/loxP*^ Myosin Cre+ mice (Fig. 3k–m). Together, these findings underscore the pivotal role of the BMAL1/HIF2A heterodimer in modulating rhythmic *AREG* induction, both under in vitro hypoxic conditions and during myocardial IRI.

## Synergistic activation of *AREG* transcription by the BMAL1/HIF2A heterodimer

**Extended Data Fig. 10.**
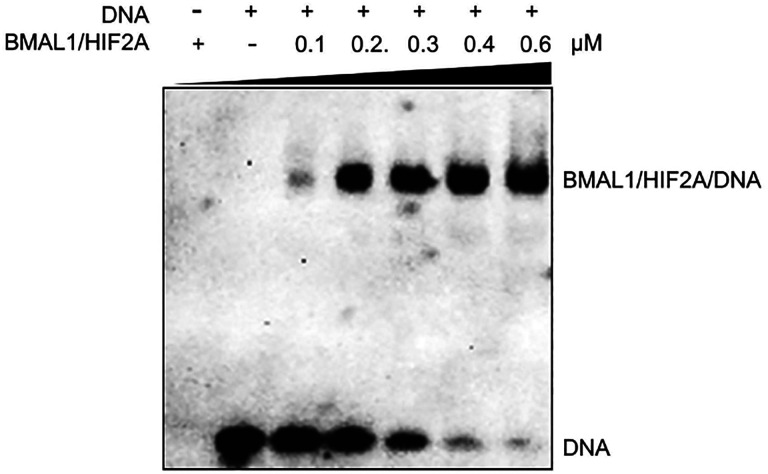
DNA-binding analysis of the BMAL1/HIF2A heterodimer. The electrophoretic mobility shift assay (EMSA) was used to investigate the binding of the BMAL1/HIF2A heterodimer to the biotinylated-HRE dsDNA. Five different protein concentrations were used, as specified. The 22 bp biotin end-labeled DNA duplex was detected using HRP-conjugated streptavidin with chemiluminescent substrates.

Next, we aimed to investigate whether the BMAL1/HIF2A heterodimer directly induces *AREG* during ambient hypoxia. Previous studies have reported that HIF2A/HIF1B and BMAL1/CLOCK complexes are capable of recognizing DNA sequences containing hypoxia response element (HRE)^62^ and E-box elements^15^, respectively, thereby initiating the transcription of target genes. Notably, these core sequences of HRE ([A/G]CGTG) and the E-box motif (CACGTG) share a striking degree of similarity. The EMSA (Extended Data Fig. 10) and surface plasmon resonance (SPR) analysis (Fig. 3n, o) provided compelling evidence of the robust and comparable binding affinity of the BMAL1/HIF2A heterodimer to both canonical HRE and E-box elements, showing the capability of BMAL1/HIF2A to recognize the DNA elements of their target genes. Subsequent examination^70^ of the human *AREG* promoter revealed the existence of multiple potential binding sites for HIF2A or BMAL1. Upon comparing these predicted binding sites, we identified a common binding site (CAGGTG) for both BMAL1 and HIF2A on the *AREG* promoter (Fig. 3p upper). Importantly, this common binding site exhibited high conservation across different species, including humans, mice, monkeys, rats, and cattle (Fig. 3p lower). Consequently, we hypothesized that both BMAL1 and HIF2A bind to this conserved common binding site to activate *AREG* transcription.

Initially, we conducted chromatin immunoprecipitation with quantitative real-time PCR (ChIP-qPCR) assays on HCMs and confirmed a significant binding enrichment of endogenous HIF2A at this common site under ambient hypoxia (1% oxygen, 4h) at CT 32 but not at CT20 (Fig. 3q). Notably, this binding enrichment pattern coincided with the increased BMAL1/HIF2A heterodimerization observed under hypoxia in the nuclear fractions of HCMs at CT32 (Fig. 3r) and the most pronounced *AREG* induction at this same window (Fig. 3g). These findings strongly suggest that the modulation of *AREG* transcription by hypoxia-induced HIF2A is gated by circadian rhythms at the chromatin level. Additional ChIP-qPCR analysis further confirmed that BMAL1 also directly binds to this common site during ambient hypoxia (Fig. 3s). However, when we tested other predicted binding sites, none demonstrated simultaneous binding by both BMAL1 and HIF2A (data not shown). To delve into the crosstalk between BMAL1 and HIF2A at the chromatin level and their joint effect on gene regulation, we cloned this sequence (CAGGTG) from human *AREG* promoter into luciferase reporter constructs and transfected these into HEK293 cells. We found that this binding site was activated by HIF2A, leading to a 4.8-fold increase in luciferase activity, and by BMAL1, with a 5.9-fold increase, when the cells were transfected with an oxygen-regulation insensitive HIF2A-HA vector (pcDNA3 *mHif2a*-P405A/P530V/N851A)^71^ or BMAL1-Flag vector (pcDNA3 *mBmal1*)^72^, respectively (Fig. 3t). This suggests that both transcription factors can functionally stimulate *AREG* transcription. Notably, when BMAL1 and HIF2A were introduced together, there was a more substantial increase in luciferase activity - over double that observed with single vector transfections (Fig. 3t). This finding highlights a synergistic effect between BMAL1 and HIF2A in driving the activation of *AREG* gene. Together, these results demonstrate a direct and cooperative role of the BMAL1/HIF2A heterodimer in regulating the rhythmic *AREG* transcription.

## AREG modulates circadian variation of myocardial injury

**Extended Data Fig. 11.**
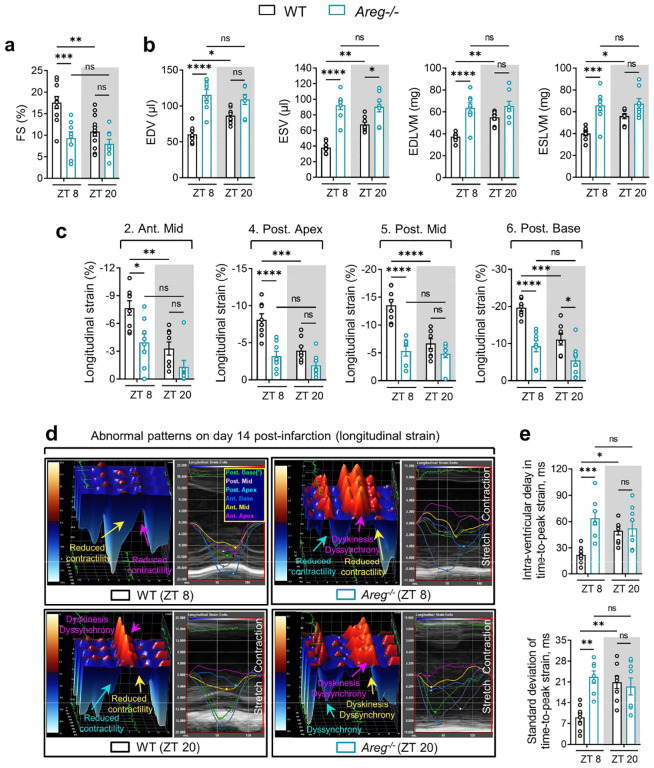
*Areg* deletion diminishes the circadian variation of cardiac function impairment. Cardiac function was measured by speckle-tracking echocardiography analysis on day 14 post-MI in *Areg*^*−/−*^ and wild-type (WT) mice subjected to IRI at ZT8 or ZT20. **a,** FS. **b,** EDV, ESV, EDLVM, and ESLVM. **c,** Left ventricular segmental wall contractility detected by peak longitudinal strain. **d,** Representative left ventricular 3D (48 points) longitudinal strain and 6-segment longitudinal strain images demonstrating wall motion abnormalities (Color-coded six segments: Ant. Base, Anterior Base; Ant. Mid, Anterior Middle; Ant. Apex, Anterior Apex; Post. Apex, Posterior Apex; Post. Mid, Posterior Middle; Post. Base, Posterior Base). **e,** Left ventricular mechanical dyssynchrony as measured by intra-ventricular delay in time-to-peak strain and standard deviation of time-to-peak strain based on longitudinal strain. n = 8/animals/group/time point. All data are mean ± s.e.m. Statistical analysis was all performed using two-way ANOVA. *p < 0.05, **p < 0.01, ***p < 0.001, and ****p < 0.0001, ns: not significant.

**Fig. 4.**
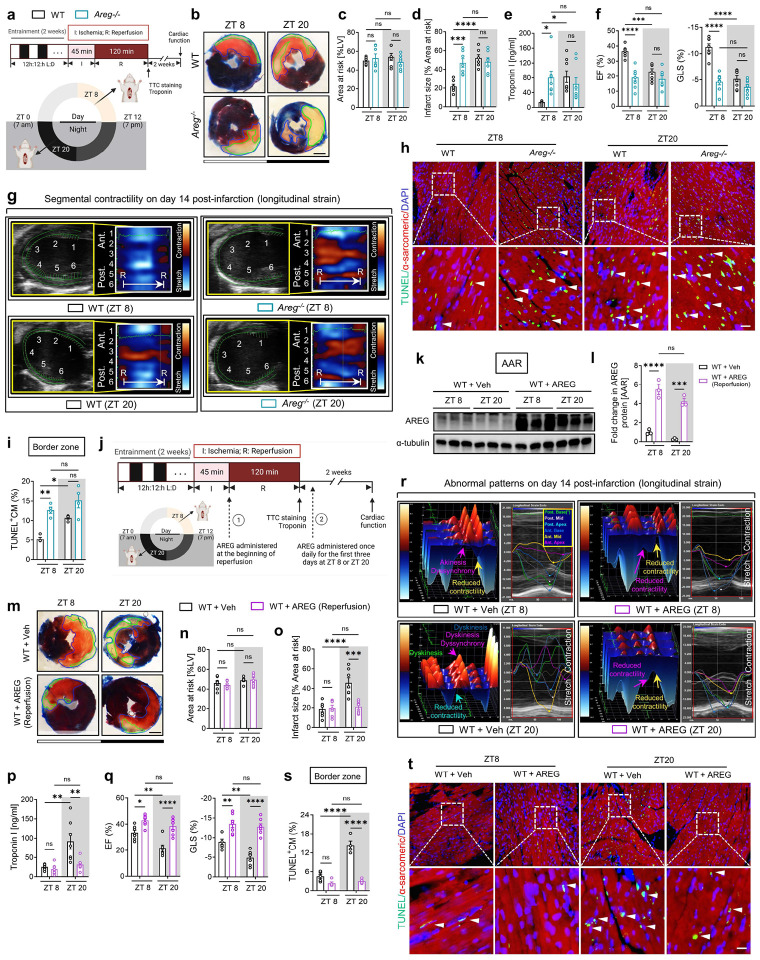
AREG drives circadian-dependent cardioprotection. **a,** Experimental setup for evaluation of myocardial injury and cardiac function in *Areg*^*−/−*^ mice and WT mice subjected to IRI at ZT8 or ZT20. **b,** Representative heart slices subjected to Evan’s blue and TTC double staining after 2 h of reperfusion: infarct area (green line) and AAR (blue line); scale bar, 1 mm. **c,** Percentage of the AAR relative to the size of the LV. n = 7 mice/group/time point. Statistical analysis was performed using two-way ANOVA. **d,** Infarct sizes represented as the percentage of the AAR. n = same as above. Statistical analysis was performed using two-way ANOVA. **e,** Serum troponin I level, evaluated at 2h after reperfusion. n = same as above. Statistical analysis was performed using two-way ANOVA. **f** and **g,** Cardiac function was evaluated by speckle-tracking echocardiography on day 14 post-MI, **f,** EF and GLS. **g,** Representative B-mode images from the left ventricular long-axis view with 2D longitudinal strain analysis, showing 6 segments (1. Ant. Base, Anterior Base; 2. Ant. Mid, Anterior Middle; 3. Ant. Apex, Anterior Apex; 4. Post. Apex, Posterior Apex; 5. Post. Mid, Posterior Middle; 6. Post. Base, Posterior Base). n = 8 mice/group/time point. Statistical analysis was performed using two-way ANOVA. **h** and **i, h,** Representative photomicrographs of TUNEL and nuclear DAPI staining of cardiomyocyte marker α-sarcomeric-positive cardiomyocytes in the border zone of hearts obtained from WT and *Areg*^*−/−*^ mice on day 1 post-MI. White arrows point out TUNEL-positive (green) cardiomyocyte (red) nuclei (blue); scale bar, 50 μm. **i,** Percentage of TUNEL-positive cardiomyocytes after MI. n = 4 mice/group/time point. Statistical analysis was performed using two-way ANOVA. **j,** Experimental setup for evaluating myocardial injury and cardiac function in AREG-treated or vehicle (Veh)-treated (saline) mice subjected to IRI at ZT8 or ZT20. To mimic the clinical scenario, AREG (10 μg) was administered at the start of reperfusion and subsequently given daily for the first three days following injury at either ZT8 or ZT20. **k,** AREG protein level was measured by Western blot analysis following 2 h of reperfusion in the AAR from AREG-treated or Veh-treated mouse hearts. n = 3 mice/group/time point. **l,** Quantification of AREG protein levels in **(k)**. n = same as above. Statistical analysis was performed using two-way ANOVA. **m,** Representative heart slices subjected to Evan’s blue and TTC double staining after 2h of reperfusion: infarct area (green line) and AAR (blue line); scale bar, 1 mm. **n,** Percentage of the AAR relative to the size of the LV. n = 7 mice/group/time point. Statistical analysis was performed using two-way ANOVA. **o,** Infarct size represented as the percentage of the AAR. n = same as above. Statistical analysis was performed using two-way ANOVA. **p,** Serum troponin I level, evaluated at 2h after reperfusion. n = same as above. Statistical analysis was performed using two-way ANOVA. **q** and **r,** Cardiac function was evaluated by speckle-tracking echocardiography analysis on day 14 post-MI in the ZT20 IRI mice following timed AREG treatment administered either at ZT8 or ZT20. **q,** EF and GLS. **r,** Representative left ventricular 3D (48 points) longitudinal strain and 6-segment longitudinal strain images demonstrating wall motion abnormalities (Color-coded six segments: Ant. Base, Anterior Base; Ant. Mid, Anterior Middle; Ant. Apex, Anterior Apex; Post. Apex, Posterior Apex; Post. Mid, Posterior Middle; Post. Base, Posterior Base). n = 7 mice/group/time point. Statistical analysis was performed using two-way ANOVA. **s** and **t, s,** Percentage of TUNEL-positive cardiomyocytes. **t,** Representative photomicrographs of TUNEL and nuclear DAPI staining of cardiomyocyte marker α-sarcomeric-positive cardiomyocytes in the border zone of hearts obtained from timed AREG-treated (ZT8 or ZT20) or Veh-treated mice on day 1 post-MI. White arrows point out TUNEL-positive (green) cardiomyocyte (red) nuclei (blue); scale bar, 50 μm. n = 5 mice/Veh-treated/ZT8, n = 4 mice/Veh-treated/ZT20, n = 5 mice/AREG-treated/ZT8, n = 5 mice/AREG-treated/ZT20. Statistical analysis was performed using two-way ANOVA. All data are mean ± s.e.m. *p < 0.05, **p < 0.01, ***p < 0.001, and ****p < 0.0001, ns: not significant.

Based on our current findings that *Areg* is a rhythmic target of the BMAL1/HIF2A heterodimer, we next investigated the influence of AREG on circadian variation of myocardial injury. Mirroring our findings in *Bmal1*^*loxP/loxP*^ Myosin Cre+ mice and *Hif2a*^*loxP/loxP*^ Myosin Cre+ mice, *Areg*^*−/−*^ mice exhibited abolished circadian dependence of myocardial injury and diminished endogenous cardioprotection at ZT8 following 2h of reperfusion, evidenced by larger infarct sizes and elevated serum troponin I levels compared to wild-type (WT) controls (Fig. 4a–e). Consistently, when subjected to IRI at ZT8, *Areg*^*−/−*^ mice experienced a pronounced decline in cardiac systolic function and markedly deteriorated long-term outcomes, including LV dilation and elevated LV mass, increased segmental wall motion abnormalities and reduced intraventricular synchronicity observed on day 14 post-MI (Fig. 4f, g and Extended Data Fig. 11). Notably, the diurnal variations of long-term myocardial injury observed in control mice completely vanished in *Areg*^*−/−*^ mice (Fig. 4f, g and Extended Data Fig. 11). Furthermore, TUNEL staining was performed to detect cardiomyocyte apoptosis on day 1 post-MI in the border zone. The proportion of TUNEL-positive cardiomyocytes showed a diminished circadian pattern, with a markedly increased rate in *Areg*^*−/−*^ mice subjected to IRI at ZT8 compared to controls (Fig. 4h, i). In contrast, differences were minimal between the ZT20 groups. Together, the exacerbated cardiac injury at ZT8 in the *Areg*-deficient mice underscores a circadian-dependent cardioprotective effect of AREG by enhancing the myocardium’s resilience against IRI at ZT8.

## Timed medication of AREG for circadian-dependent cardioprotection

**Extended Data Fig. 12.**
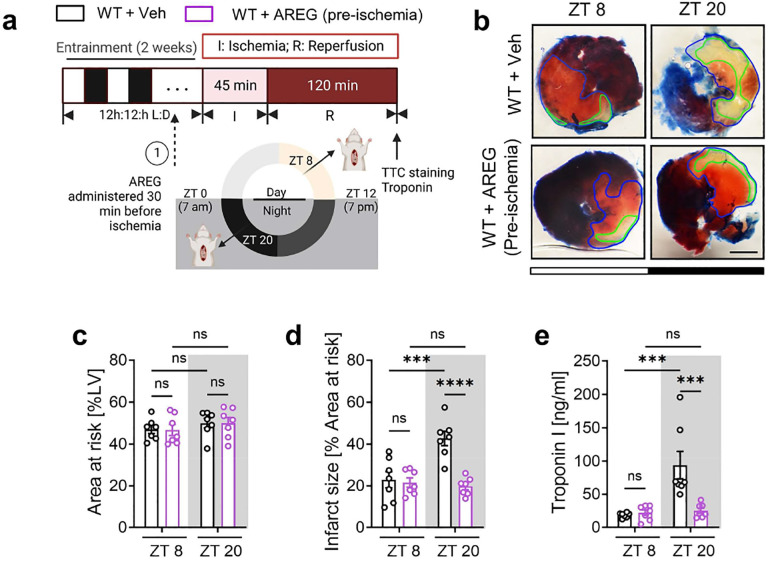
AREG administered before ischemia dampens the myocardial injury at ZT20. **a,** Schematic of the experimental setup for evaluating myocardial injury in AREG-treated or vehicle (Veh)-treated (saline) mice subjected to myocardial IRI at ZT8 or ZT20. AREG (10 μg) was administered 30 minutes before IRI. **b,** Representative heart slices subjected to Evan’s blue and TTC double staining after 2h of reperfusion: infarct area (green line) and AAR (blue line); scale bar, 1 mm. **c,** Percentage of the AAR relative to the size of the LV. **d,** Infarct sizes represented as the percentage of the AAR. **e,** Serum troponin I levels. n = 7 mice/group/time point. All data are mean ± s.e.m. Statistical analysis was performed using two-way ANOVA. ***p < 0.001, and ****p < 0.0001, ns: not significant.

**Extended Data Fig. 13.**
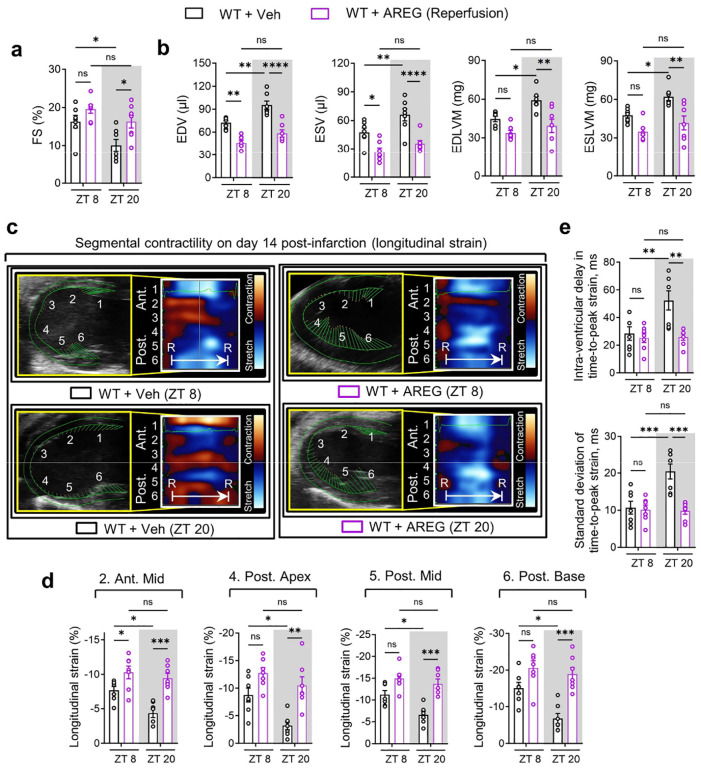
Timed AREG treatment improves cardiac function when administered at ZT20. Cardiac function was evaluated in timed AREG treatment or Vehicle treatment (saline) in mice subjected to IRI at ZT20. To mimic the clinical scenario where treatment begins after myocardial infarction onset, AREG (10 μg) or vehicle was administered at the start of reperfusion and subsequently given daily post-MI at either ZT8 or ZT20. **a,** FS. **b,** EDV, ESV, EDLVM and ESLVM. **c,** Representative B-mode images from the left ventricular long-axis view with 2D longitudinal strain analysis, showing 6 segments (1. Ant. Base, Anterior Base; 2. Ant. Mid, Anterior Middle; 3. Ant. Apex, Anterior Apex; 4. Post. Apex, Posterior Apex; 5. Post. Mid, Posterior Middle; 6. Post. Base, Posterior Base). **d,** Left ventricular segmental wall contractility detected by peak longitudinal strain. **e,** Left ventricular mechanical dyssynchrony as measured by intra-ventricular delay in time-to-peak strain and standard deviation of time-to-peak strain based on longitudinal strain. n = 7 mice/group/time point. Data are mean ± s.e.m. Statistical analysis was performed using two-way ANOVA. *p < 0.05, **p < 0.01, ***p < 0.001, and ****p < 0.0001, ns: not significant.

To further explore the potential of AREG as a circadian-dependent cardioprotective target, we first administered a single dose of mouse recombinant AREG protein (10 μg) or a vehicle control via the carotid artery to C57BL/6J mice and subjected them to myocardial IRI at either ZT8 or ZT20 (Extended Data Fig. 12a). This approach markedly alleviated myocardial injury, as shown by smaller infarct sizes and lower serum troponin I levels following 2h of reperfusion (Extended Data Fig. 12b–e). Interestingly, these cardioprotective effects were more pronounced at ZT20, which we suspect may be due to the diurnal pattern of endogenous *AREG* induction in ischemic mouse hearts, with a trough at ZT20 (Fig. 3b–f). Thus, AREG administration at this time (ZT20) appeared to be more beneficial. To simulate the clinical scenario where treatment is initiated after the onset of MI, we administered AREG (10 μg) at the start of reperfusion (Fig. 4j) and observed markedly elevated AREG protein levels in the AAR of mouse hearts at both time points (Fig. 4k, l). This intervention mirrored our previous findings, reinforcing the circadian-dependent cardioprotective effects of AREG administration at ZT20 following 2h of reperfusion (Fig. 4m–p). To further investigate the long-term effects of timed AREG on cardiac function, we administered AREG (10 μg) daily for the first three days following injury either at ZT8 or ZT20 in C57BL/6J mice subjected to myocardial IRI at ZT20 (Fig. 4j). By day 14 post-MI, STE analysis revealed that AREG administration, especially at ZT20, improved LV systolic function (increased EF, FS and GLS) and mitigated long-term myocardial injury (reduced LV chamber dilation and LV mass) (Fig. 4q and Extended Data Fig. 13a, b). Moreover, AREG administration at ZT20 significantly restored segmental contractility (Extended Data Fig. 13c, d) and re-introduced normokinesis by reducing abnormal wall motion patterns and intra-ventricular dyssynchrony (Fig. 4r and Extended Data Fig. 13e). Furthermore, TUNEL staining on day 1 post-MI revealed notably reduced cardiomyocyte apoptosis in the border zone of mice treated with AREG at ZT20 (Fig. 4s, t). These findings suggest that AREG treatment provides circadian-dependent cardioprotection, particularly when administered in alignment with the endogenous trough at ZT20 (Fig. 3b–f).

## Pharmacologically targeting BMAL1 using nobiletin for circadian-dependent cardioprotection.

**Extended Data Fig. 14.**
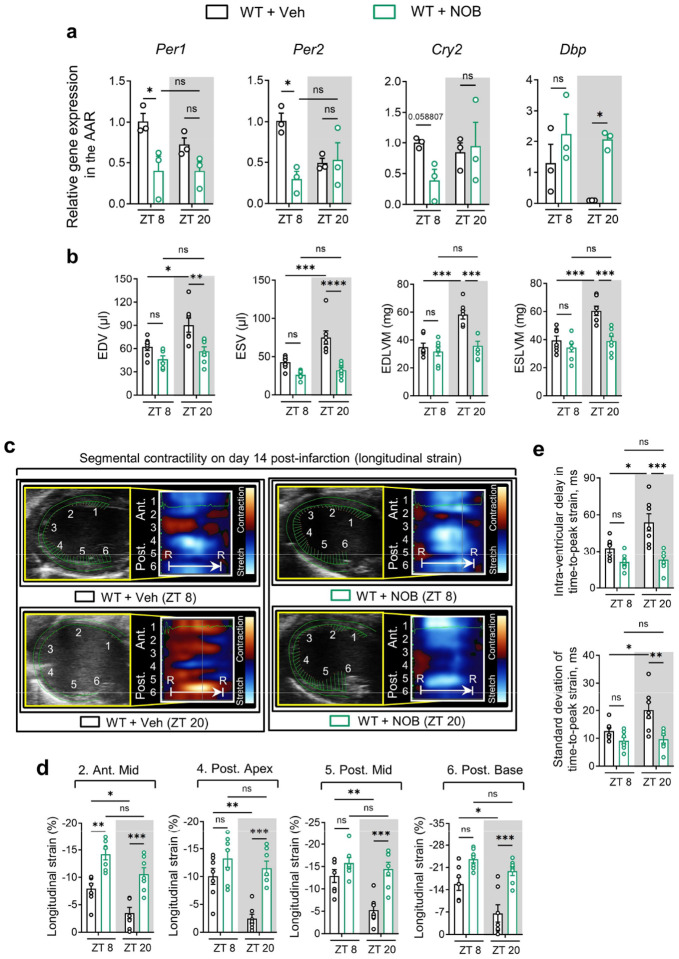
NOB treatment provides circadian-dependent cardioprotection at ZT20. NOB-treated (200 mg/kg, i.p., every other day) or vehicle-treated C57BL/6J mice were subjected to myocardial IRI at ZT8 or ZT20. **a,** Transcript levels of *Per1*, *Per2*, *Cry2*, and *Dbp* in the AAR from mouse hearts were measured by real-time PCR analysis following 2h of reperfusion. n = 3 mice/group/time point. **b-e.** Cardiac function was evaluated by speckle-tracking echocardiography analysis on day 14 post-MI. **b,** EDV, ESV, EDLVM and ESLVM. **c,** Representative B-mode images from the left ventricular long-axis view with 2D longitudinal strain analysis, showing 6 segments (1. Ant. Base, Anterior Base; 2. Ant. Mid, Anterior Middle; 3. Ant. Apex, Anterior Apex; 4. Post. Apex, Posterior Apex; 5. Post. Mid, Posterior Middle; 6. Post. Base, Posterior Base). **d,** Left ventricular segmental wall contractility detected by peak longitudinal strain. **e,** Left ventricular mechanical dyssynchrony as measured by intra-ventricular delay in time-to-peak strain and standard deviation of time-to-peak strain based on longitudinal strain. b-e: n = 7 mice/group/time point. Data are mean ± s.e.m. Statistical analysis was performed using two-way ANOVA. *p < 0.05, **p < 0.01, ***p < 0.001, and ****p < 0.0001, ns: not significant.

**Fig. 5.**
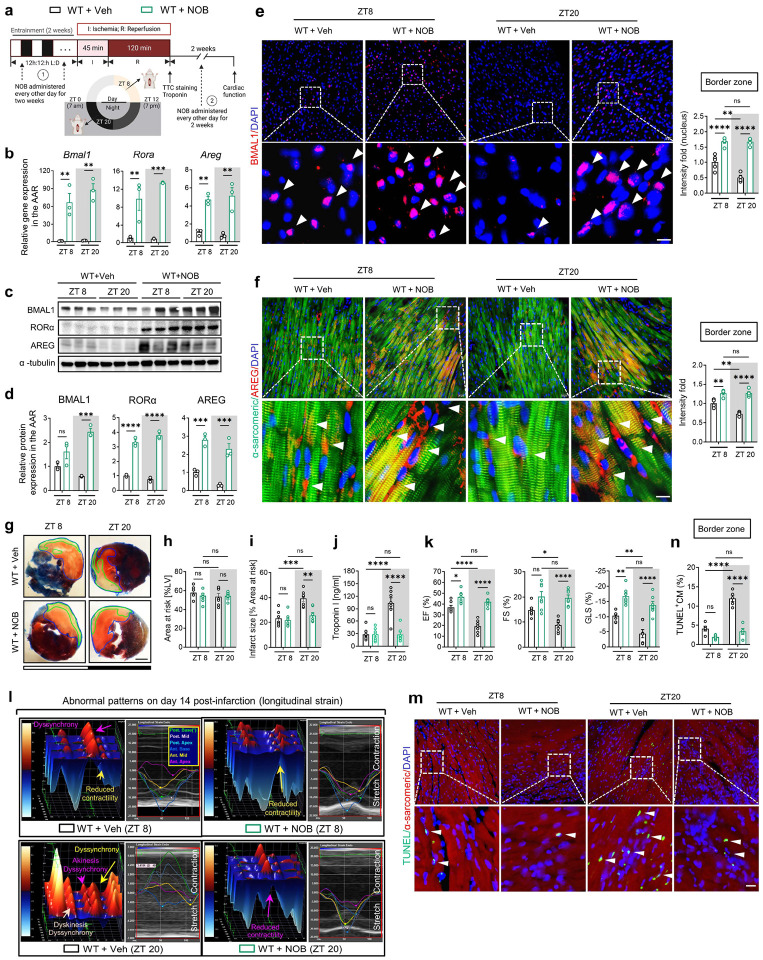
Targeting BMAL1 using NOB for circadian-dependent cardioprotection. **a,** Experimental setup for evaluating myocardial injury and cardiac function in NOB-treated (200 mg/kg, i.p., every other day) or Veh-treated C57BL/6J mice subjected to myocardial IRI at ZT8 or ZT20. **b-d,** Transcript **(b)** and protein levels **(c)** of BMAL1, RORα and AREG were measured following 2h of reperfusion in the AAR from NOB-treated or Veh-treated C57BL/6J mouse hearts. **d,** Quantification of protein levels in **(c).** n = 3 mice/group/time point. Statistical analysis was performed using two-way ANOVA. **e,** Representative images, along with quantification of BMAL1 immunofluorescence staining (red) in cell nuclei (DAPI: blue), on day 1 following IRI in the border zone of hearts from NOB-treated or Veh-treated mice subjected to myocardial IRI at ZT8 or ZT20; Scale bar, 50 μm. White arrows indicate BMAL1 within the nuclei. n = 5 mice/Veh-treated/ZT8, n = 4 mice/Veh-treated/ZT20, n = 5 mice/NOB-treated/ZT8, and n = 5 mice/NOB-treated/ZT20. Statistical analysis was performed using two-way ANOVA. **f,** Representative immunostaining of AREG (red), α-sarcomeric (green), and nuclei (DAPI: blue) on day 1 post-MI in the border zone of hearts from NOB-treated or Veh-treated mice subjected to myocardial IRI at ZT8 or ZT20; Scale bar, 50 μm. Arrows indicate α-sarcomeric^+^/AREG^+^ cells. The quantification of the fold change in AREG intensity is also presented. n = 4 mice/Veh-treated/ZT8, n = 5 mice/Veh-treated/ZT20, n = 4 mice/NOB-treated/ZT8, and n = 5 mice/NOB-treated/ZT20. Statistical analysis was performed using two-way ANOVA. **g,** Representative heart slices subjected to Evan’s blue and TTC double staining after 2h of reperfusion: infarct area (green line) and AAR (blue line); scale bar, 1 mm. **h,** Percentage of the AAR relative to the size of the LV. n = 7 mice/group/time point. Statistical analysis was performed using two-way ANOVA. **i,** Infarct size represented as the percentage of the AAR. n = same as above. Statistical analysis was performed using two-way ANOVA. **j,** Serum troponin I levels were evaluated at 2h after reperfusion. n = same as above. Statistical analysis was performed using two-way ANOVA. **k** and **l,** Cardiac function was evaluated by speckle-tracking echocardiography on day 14 post-MI in NOB-treated or Veh-treated mice subjected to myocardial IRI at ZT8 or ZT20. **k,** EF, FS, and GLS. **l,** Representative left ventricular 3D (48 points) longitudinal strain and 6-segment longitudinal strain images demonstrating wall motion abnormalities (Color-coded six segments: Ant. Base, Anterior Base; Ant. Mid, Anterior Middle; Ant. Apex, Anterior Apex; Post. Apex, Posterior Apex; Post. Mid, Posterior Middle; Post. Base, Posterior Base). n = 7 mice/group/time point. Statistical analysis was performed using two-way ANOVA. **m** and **n, m,** Representative photomicrographs of TUNEL and nuclear DAPI staining of cardiomyocyte marker α-sarcomeric-positive cardiomyocytes in the border zone of hearts obtained from Veh-treated or NOB-treated mice on day 1 post-MI. White arrows point out TUNEL-positive (green) cardiomyocyte (red) nuclei (blue); scale bar, 50 μm. **n,** Percentage of TUNEL-positive cardiomyocytes in **(m)**. n = 5 mice/group/time point. Statistical analysis was performed using two-way ANOVA. All data are mean ± s.e.m.*p < 0.05, **p < 0.01, ***p < 0.001, and ****p < 0.0001, ns: not significant.

Based on our current observations of the direct interplay between circadian rhythm (BMAL1) and hypoxia signaling (HIF2A) in modulating the circadian variation of myocardial injury, we next explored the therapeutic potential of targeting circadian rhythm (BMAL1) for treating myocardial injury. Nobiletin (NOB), a flavonoid compound naturally abundant in citrus fruit peels, has been demonstrated to enhance circadian rhythms in vitro and in vivo^73–75^. Initially, we administered NOB (200 mg/kg, i.p.) to C57BL/6J mice during their active period (ZT14–20) every other day for two weeks and subjected them to myocardial IRI at either ZT8 or ZT20 (Fig. 5a). While NOB doesn’t target BMAL1 directly, it activates key transcription factors in the molecular clock: the retinoic acid receptor-related orphan receptors (RORs)^73^. These RORs can bind to ROR response elements (ROREs) in the *Bmal1* promoter region, stimulating its transcription^14,19,37,76,77^. Our findings supported this regulatory mechanism, with a marked rise in RORα expression and a striking 60–80-fold increase in *Bmal1* transcript levels, along with a 2–3-fold surge in protein levels after NOB treatment (Fig. 5b–d). In addition, NOB treatment led to a reduction in the expression of clock genes within the negative feedback loop, such as *Per1*, *Per2*, and *Cry2*, at ZT8 (Extended Data Fig. 14a). Concurrently, we observed a marked increase in the levels of *Dbp*, a target gene of BMAL1, at ZT20 in the mouse heart (Extended Data Fig. 14a). This indicates an enhancement in the transcriptional activity of BMAL1 at ZT20, stemming from the mitigation of suppression by the negative feedback loop. Consequently, we observed a significant increase in both the transcript and protein levels of AREG (Fig. 5b–d). Notably, immunofluorescence staining also revealed an increase in nuclear BMAL1 and cytoplasmic AREG expressions in cardiomyocytes in the border zone of ischemic mouse hearts on day 1 post-MI (Fig. 5e, f). Taken together, these results highlight NOB’s capability to regulate the circadian rhythms and boost the BMAL1-AREG pathway.

**Extended Data Fig. 15.**
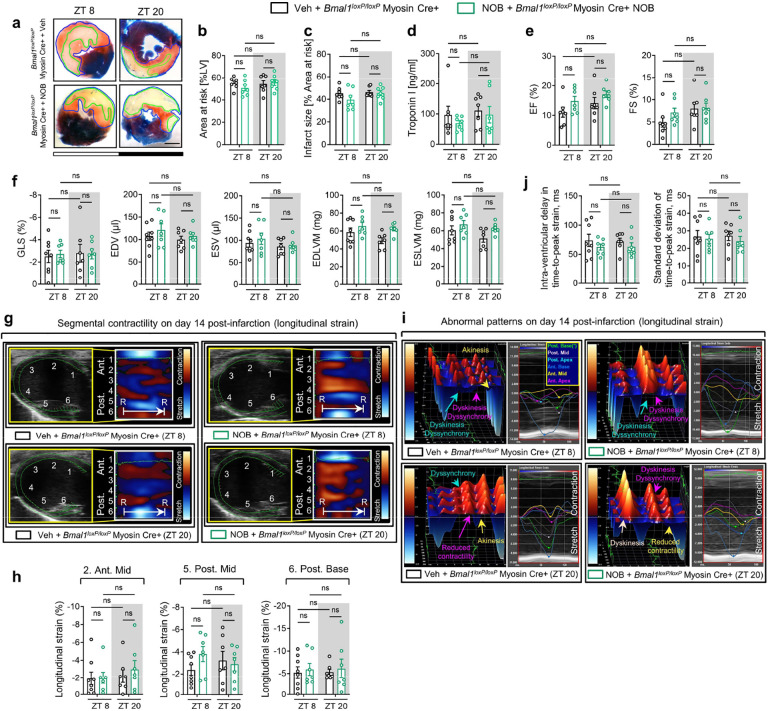
NOB failed to alleviate myocardial injury in *Bmal1*^*loxP/loxP*^ Myosin Cre+ mice. NOB-treated (200 mg/kg, i.p., every other day) or Veh-treated *Bmal1*^*loxP/loxP*^ Myosin Cre+ mice were subjected to myocardial IRI at ZT8 or ZT20. **a-d, a,** Representative heart slices subjected to Evan’s blue and TTC double staining after 2 h of reperfusion: infarct area (green line) and AAR (blue line); scale bar, 1 mm. **b,** Percentage of the AAR relative to the size of the LV. **c,** Infarct sizes represented as the percentage of the AAR. **d,** Serum troponin I levels. n = 7 mice/group/time point. **e-j,** Cardiac function was evaluated by speckle-tracking echocardiography analysis on day 14 post-MI. **e,** EF and FS. **f,** GLS, EDV, ESV, EDLVM, and ESLVM. **g,** Representative B-mode images from the left ventricular long-axis view with 2D longitudinal strain analysis, showing 6 segments (1. Ant. Base, Anterior Base; 2. Ant. Mid, Anterior Middle; 3. Ant. Apex, Anterior Apex; 4. Post. Apex, Posterior Apex; 5. Post. Mid, Posterior Middle; 6. Post. Base, Posterior Base). **h,** Left ventricular segmental wall contractility detected by peak longitudinal strain. **i,** Representative left ventricular 3D (48 points) longitudinal strain and 6-segment longitudinal strain images demonstrating wall motion abnormalities. **j,** Left ventricular mechanical dyssynchrony as measured by intra-ventricular delay in time-to-peak strain and standard deviation of time-to-peak strain based on longitudinal strain. n = 8/Veh-treated *Bmal1*^*loxP/loxP*^ Myosin Cre+/time point, n = 7/NOB-treated *Bmal1*^*loxP/loxP*^ Myosin Cre+/time point. All data are mean ± s.e.m. Statistical analysis was all performed using two-way ANOVA. ns: not significant.

**Extended Data Fig. 16.**
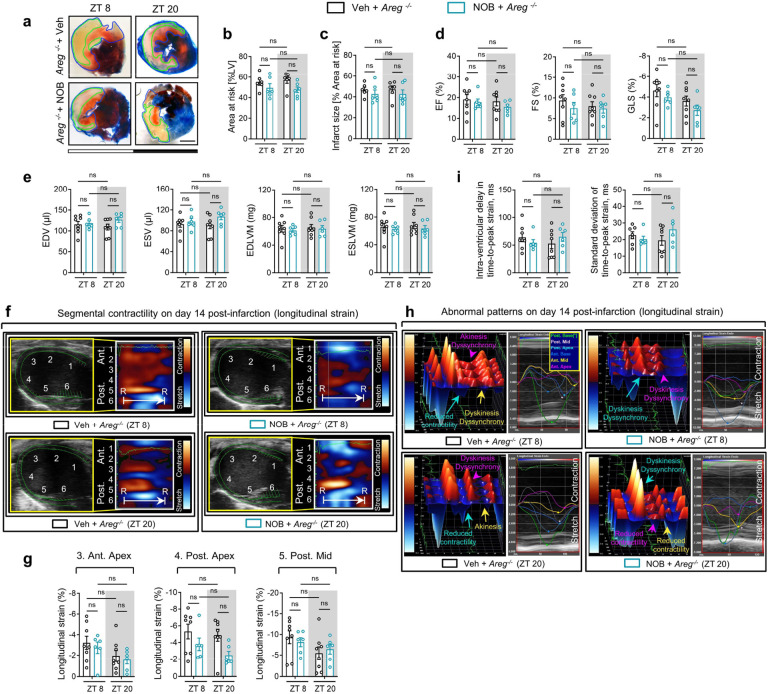
NOB failed to alleviate myocardial injury in *Areg*^*−/−*^ mice. NOB-treated (200 mg/kg, i.p., every other day) or Veh-treated *Areg*^*−/−*^ mice were subjected to myocardial IRI at ZT8 or ZT20. **a-c, a,** Representative heart slices subjected to Evan’s blue and TTC double staining after 2h of reperfusion: infarct area (green line) and AAR (blue line); scale bar, 1 mm. **b,** Percentage of the AAR relative to the size of the LV. **c,** Infarct sizes represented as the percentage of the AAR (n = 6 animals/group/time point). **d-i** Cardiac function was evaluated by speckle-tracking echocardiography analysis on day 14 post-MI. **d,** EF, FS, GLS. **e,** EDV, ESV, EDLVM, and ESLVM. **f,** Representative B-mode images from the left ventricular long-axis view with 2D longitudinal strain analysis, showing 6 segments (1. Ant. Base, Anterior Base; 2. Ant. Mid, Anterior Middle; 3. Ant. Apex, Anterior Apex; 4. Post. Apex, Posterior Apex; 5. Post. Mid, Posterior Middle; 6. Post. Base, Posterior Base). **g,** Left ventricular segmental wall contractility detected by peak longitudinal strain. **h,** Representative left ventricular 3D (48 points) longitudinal strain and 6-segment longitudinal strain images demonstrating wall motion abnormalities. **i,** Left ventricular mechanical dyssynchrony as measured by intra-ventricular delay in time-to-peak strain and standard deviation of time-to-peak strain based on longitudinal strain. n = 8/Veh-treated *Areg*^*−/−*^/time point and n = 6/NOB-treated *Areg*^*−/−*^/time point. Data are mean ± s.e.m. Statistical analysis was performed using two-way ANOVA. ns: not significant.

We then examined the impact of NOB treatment on myocardial injury. Following 2h of reperfusion, mice subjected to IRI at ZT20 and treated with NOB exhibited a marked alleviation of myocardial injury, characterized by smaller infarct sizes and reduced serum troponin I levels following 2h of reperfusion (Fig. 5g–j). On the contrary, NOB treatment showed no significant cardioprotection in mice subjected to IRI at ZT8 (Fig. 5g–j). These circadian-dependent cardioprotective effects are likely linked to the inherent trough of nuclear BMAL1 expression and the concurrent dip in its transcriptional activity observed at ZT20^19,20,37,77^, which is effectively compensated by NOB treatment, resulting in pronounced cardioprotection at this time point. To further assess the long-term effects of NOB on cardiac function and remodeling, we continued to administer NOB (200 mg/kg, i.p.) every other day following IRI (Fig. 5a). On day 14 post-MI, NOB treatment led to improved LV systolic function (increased EF, FS, and GLS), and reduced LV dilation and LV mass compared to the vehicle-treated controls (Fig. 5k and Extended Data Fig. 14b). Moreover, NOB displayed potential in mitigating segmental wall motion abnormalities (Fig. 5l and Extended Data Fig. 14c, d) and attenuating intra-ventricular dyssynchrony, thereby promoting global cardiac resynchronization (Fig. 5l and Extended Data Fig. 14e). Echoing the results from the acute phase, these long-term cardiac benefits were predominantly detected in mice subjected to IRI at ZT20, further emphasizing the time-of-day-dependent cardioprotection by NOB. TUNEL staining further confirmed the circadian-dependent cardioprotection provided by NOB treatment, demonstrating greater efficiency in reducing apoptotic cardiomyocytes in the ZT20 IRI mouse group (Fig. 5n, m). Finally, we investigated whether the cardioprotective effects of NOB are dependent on BMAL1 and its downstream target, *AREG*. Intriguingly, NOB failed to alleviate myocardial injury in *Bmal1*^*loxP/loxP*^ Myosin Cre+ mice (Extended Data Fig. 15) and in *Areg*^*−/−*^ mice (Extended Data Fig. 16). Together, these results strongly suggest that NOB’s cardioprotective effects are mediated by the BMAL1-AREG signaling pathway.

## Unveiling BMAL1/HIF2A transactivation capability through DNA-bound cryo-EM structure

**Extended Data Fig. 17.**
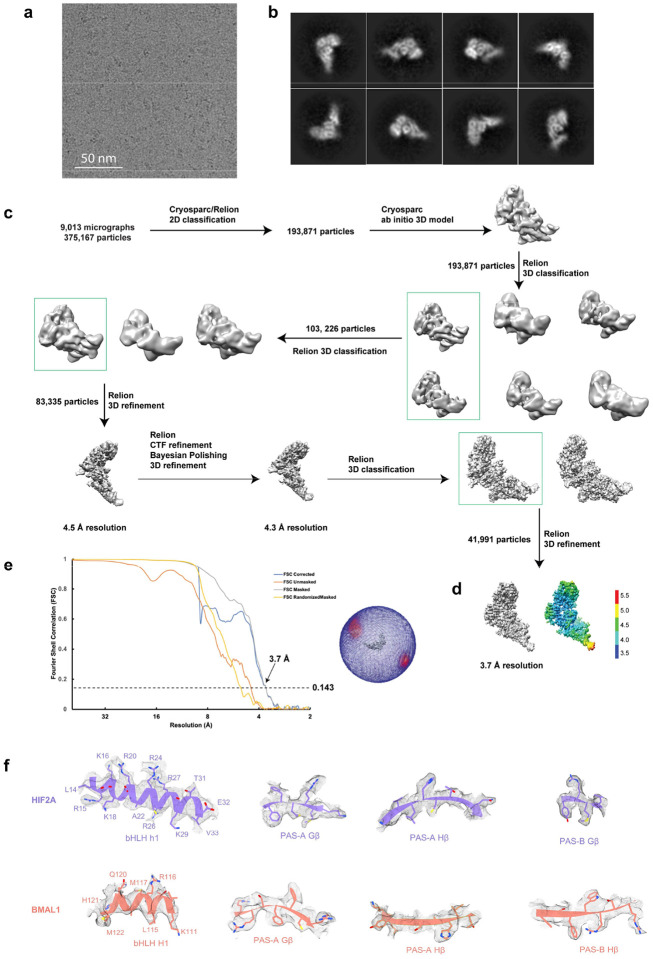
Cryo-EM structure determination of the BMAL1/HIF2A/DNA complex. **a,** A cryo-EM micrograph of the BMAL1/HIF2A/DNA complex. **b,** Representative 2D class averages of the BMAL1/HIF2A/DNA complex. **c,** Flowchart of image processing of the BMAL1/HIF2A/DNA cryo-EM data. **d,** Cryo-EM density map of the BMAL1/HIF2A/DNA complex colored by local resolution. **e,** FSC curves (left) and angular distribution plot (right) of the BMAL1/HIF2A/DNA complex. The average resolution for the BMAL1/HIF2A/DNA complex is 3.7 Å according to the gold-standard FSC = 0.143 criterion. **f,** Local structures with their corresponding densities from bHLH, PAS-A and PAS-B domains are shown.

**Fig. 6.**
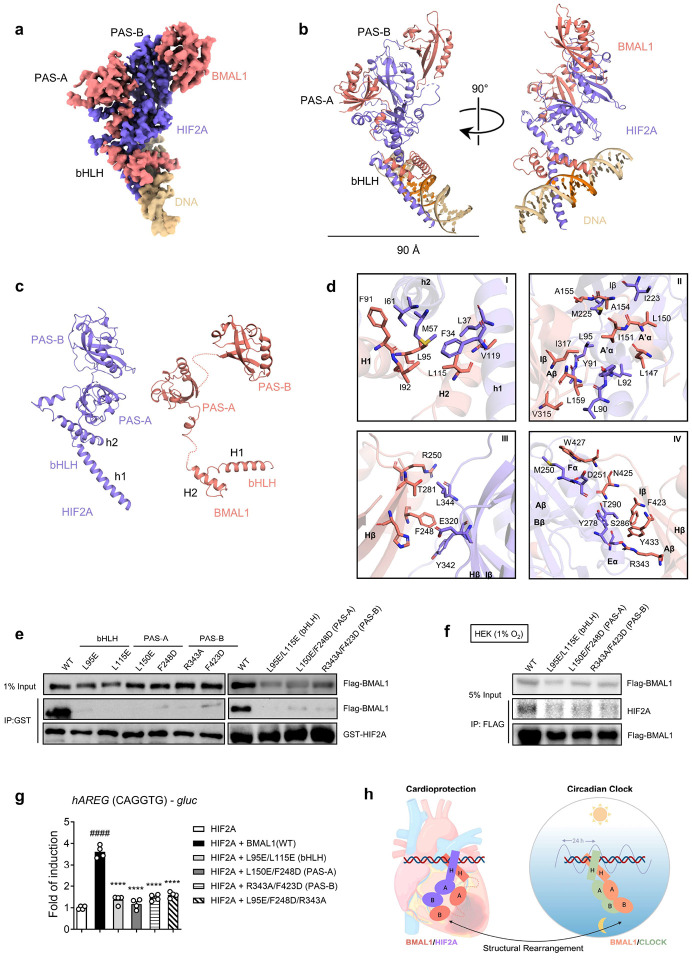
Structural analysis of the BMAL1/HIF2A heterodimer in complex with DNA. **a,** Cryo-EM density map of the BMAL1/HIF2A/DNA complex. The HIF2A, BMAL1, and HRE DNA are colored in purple, red, and yellow, respectively. The bHLH, PAS-A, and PAS-B domains are as indicated. **b,** Two views of the overall structure of the BMAL1/HIF2A/DNA complex. **c,** Individual structures of HIF2A and BMAL1 within the DNA-bound BMAL1/HIF2A heterodimer. **d,** Detailed interactions of the four interfaces (I to IV) between HIF2A and BMAL1. Residues involved in the interaction between BMAL1 (red) and HIF2A (purple) are indicated. **e,** Pull-down analysis showing impaired interaction of GST-HIF2A with Flag-tagged BMAL1 mutants. Mutations in bHLH, PAS-A, and PAS-B domains of the BMAL1 are indicated. n = 3 independent experiments. **f,** HEK293 cells overexpressing WT or mutated Flag-tagged BMAL1 were exposed to ambient hypoxia (1% O_2_) for 4h, then followed by immunoprecipitation with Flag and blotted with anti-HIF2A and anti-Flag antibodies. n = 3 independent experiments. **g,** HEK293 cells were transfected with either WT or mutated BMAL1, along with the HIF2A vector. Luciferase reporter assay was conducted to evaluate the transcription activation activity of the BMAL1/HIF2A complex on human *AREG*, which contains the common binding site. n = 4 independent experiments. Data are mean ± s.e.m. Statistical analysis was performed using one-way ANOVA. ####p < 0.0001 compared to cells transfected with HIF2A only, ****p < 0.0001 compared to cells transfected with WT BMAL1. **h,** Schematic representation illustrating the substantial structural rearrangement of BMAL1 (red) when accommodating various partners to be involved in different pathways. The PAS domains of BMAL1 (red) bend toward nearly opposite direction and position separately when intertwining with HIF2A (purple).

Based on the above studies demonstrating a functional role by which the BMAL1/HIF2A heterodimer mediates the circadian variation of myocardial injury through the rhythmic regulation of AREG expression, we next pursued studies to provide a detailed molecular description of the interaction between BMAL1 and HIF2A. While previous studies have reported the interaction between BMAL1 and HIF2A^67,78,79^, detailed structural information regarding their heterodimerization and DNA binding mode has yet to be defined. To gain a molecular understanding of heterodimerization between BMAL1 and HIF2A, as well as their DNA recognition, we successfully determined the structure of their complex bound to DNA containing a canonical HRE element using single-particle cryo-EM (Extended Data Fig. 17a and Extended Data Table 3). After 2D and 3D classification, the final ~40,000 polished particles were subjected to 3D reconstruction and refinement (Extended Data Fig. 17b, c). The final refined density map, with an average 3.7 Å resolution, reveals a 22-bp DNA duplex bound by the monomeric heterodimer (Fig. 6a and Extended Data Fig. 17d, e). An atomic model of the DNA-bound heterodimer was built into the density map using the crystal structures of BMAL1 and HIF2A from BMAL1/CLOCK (PDB: 4F3L)^15^ and HIF2A/HIF1B (PDB: 4ZP4)^62^ complexes as templates, respectively (Extended Data Fig. 17f).

**Extended Data Fig. 18.**
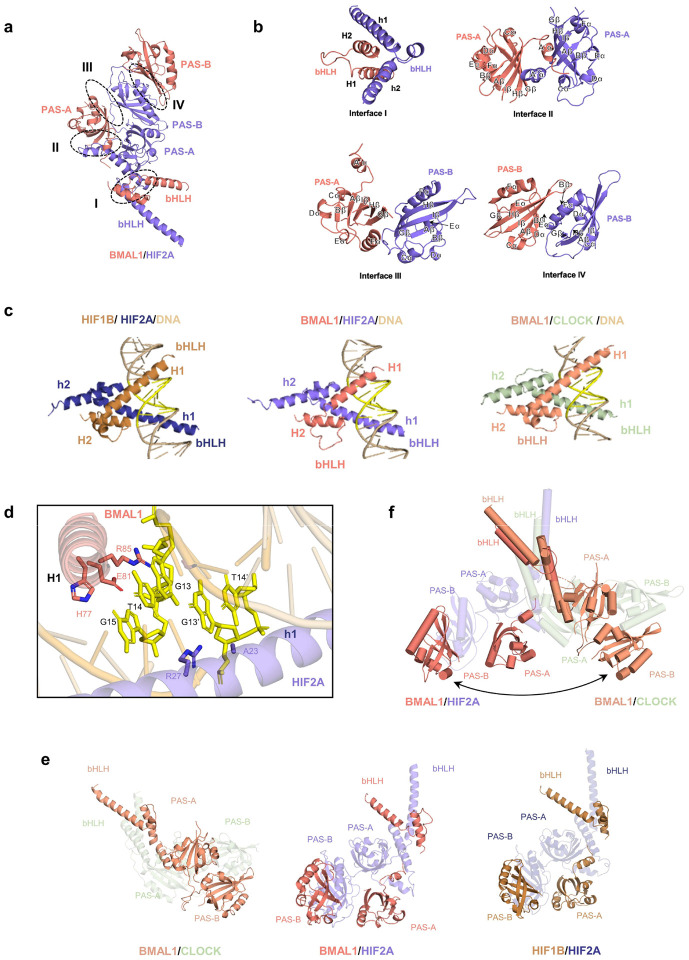
Structural analysis of the BMAL1/HIF2A/DNA complex. **a,** Four domain interfaces (I to IV) between BMAL1 and HIF2A. Each of these is indicated by a dashed ellipse. **b,** Zoom-in views of the interfaces (I to IV) between HIF2A and BMAL1. **c,** Comparison of the DNA-binding by two bHLH domains in HIF1B/HIF2A (left, PDB ID 4ZPK), BMAL1/HIF2A (middle), and BMAL1/CLOCK (right, PDB ID 4H10). The DNA contacted by the bHLH domains is highlighted in yellow. The PAS domains are omitted for clarity. **d,** Detailed interactions between two bHLH domains of BMAL1/HIF2A and the HRE DNA (yellow). **e,** Structural comparison of BMAL1/CLOCK (left, PDB ID 4F3L), BMAL1/HIF2A (middle), and HIF1B/HIF2A (right, PDB ID 4ZPK). For clarity, DNA was omitted. **f,** BMAL1 undergoes structural rearrangements upon binding with various partners. Superimposing the BMAL1/HIF2A and BMAL1/CLOCK complexes by aligning their bHLH domains reveals that BMAL1 undergoes a substantial conformational change, with the two PAS domains bending in nearly opposite directions. BMAL1 exhibits a compact overall architecture when bound with CLOCK (green) and a distinctly separated conformation when interacting with HIF2A (purple).

In the complex structure, both BMAL1 and HIF2A exhibit distinct architectures while binding to DNA through their bHLH domains (Fig. 6b). HIF2A adopts an elongated and compact conformation in which the PAS-A domain serves as a bridge between the bHLH and PAS-B domains (Fig. 6c left). In contrast, the bHLH and PAS domains of BMAL1 are arranged separately without establishing any inter-domain contacts (Fig. 6c right). To dimerize with HIF2A, the BMAL1 utilizes its bHLH and PAS domains to wrap around their respective counterparts in HIF2A, resulting in the establishment of four major interfaces (I to IV) characterized by extensive intermolecular contacts (Fig. 6d and Extended Data Fig. 18a, b). Upon DNA binding, the BMAL1/HIF2A heterodimer primarily utilizes its two bHLH domains to recognize the HRE element, employing a DNA binding mode similar to the modes observed in both HIF1B/HIF2A^62^ and BMAL1/CLOCK^80^ structures (Extended Data Fig. 18c). Specifically, two basic α-helices, H1 and h1, within the bHLH domains of BMAL and HIF2A, respectively, insert into the major groove of the HRE element, establishing multiple contacts for base recognition (Extended Data Fig. 18d).

Interestingly, the structure of BMAL1 bound to HIF2A differs significantly from that of its complex with CLOCK^15^, in which the PAS domains of BMAL1 are tightly packed together (Extended Data Fig. 18e). Conversely, in the BMAL1/HIF2A structure, the PAS domains of BMAL1 are positioned separately and arranged in a conformation similar to the HIF1B observed in its complex with HIF2A (Extended Data Fig. 18e). Additionally, by superimposing the bHLH domain of BMAL1 present in both the BMAL1/CLOCK and BMAL1/HIF2A complexes, we observed that the PAS domains of BMAL1 bend toward nearly opposite directions, indicating that BMAL1 undergoes a significant conformational change when accommodating its different partners (Extended Data Fig. 18f and Supplementary Video1).

The intermolecular contacts between BMAL1 and HIF2A involve several conserved hydrophobic and polar residues (Fig. 6d). To validate our structural observations, we generated BMAL1 mutants and conducted GST pull-down assays to examine their interactions with HIF2A. We observed that introducing single and double mutations within the bHLH and PAS domains of BMAL1 significantly impaired the formation of the BMAL1/HIF2A complex (Fig. 6e). Additionally, these mutants disrupted the interaction between BMAL1 and endogenous HIF2A in HEK293 cells during ambient hypoxia (1% oxygen, 4h) (Fig. 6f). These results demonstrated that interactions mediated by the bHLH, PAS-A, and PAS-B domains collectively contribute to the stability of this heterodimeric complex. To further examine the impact of complex disruption on their ability to regulate *AREG* induction, we performed a luciferase reporter assay using HEK293 cells transfected with these mutants along with an oxygen-regulation insensitive HIF2A-HA vector (pcDNA3 *mHif2a*-P405A/P530V/N851A)^71^ and the human *AREG*-g*luc* reporter harboring the common binding site sequence (CAGGTG). We found that the BMAL1 mutants significantly reduced the transcription activation activity of the BMAL1/HIF2A complex at the *AREG* promoter compared to the WT control, indicating that efficient *AREG* transactivation relies on the proper heterodimerization of the BMAL1/HIF2A complex (Fig. 6g).

Collectively, our study provides structural evidence that confirms the formation of the BMAL1/HIF2A heterodimer and its ability to recognize DNA, thus further reinforcing the functional role of BMAL1/HIF2A in regulating its target genes, such as *AREG*. Moreover, our structure highlights the capability of BMAL1 to bridge various pathways through significant structural rearrangements required for binding to different partners (Fig. 6h).

## Discussion

Acute injury and long-term outcomes of myocardial infarction exhibit a circadian pattern^12–14,31,56,81^, yet the underlying mechanisms have remained elusive. The current studies newly identified a molecular mechanism governing this phenomenon: BMAL1 directly interacts with HIF2A to form a transcriptionally active heterodimer, which drives the circadian variation of myocardial injury in both the acute and long-term phases in mice post-MI. Our biophysical and biochemical studies further provide the structural basis for understanding how BMAL1 and HIF2A interact and recognize DNA. Intriguingly, we identify *Areg* as a rhythmic transcriptional target gene of the BMAL1/HIF2A heterodimer in determining the diurnal variation of cardiac injury. Leveraging this knowledge, we demonstrate that timed administration of AREG or pharmacologically targeting BMAL1 using NOB can significantly alleviate myocardial injury and improve cardiac function in a time-of-day-dependent manner.

In cardiomyocytes, the core circadian transcription factor BMAL1 is implicated in various aspects of cardiac health and diseases, including mitochondrial function^51,52,82^, heart development^59^, contractile function^51,52^, cell death^36^, and heart failure^51,55,56^. Despite this extensive involvement, a significant knowledge gap has persisted regarding its functional roles in the diurnal variation of myocardial injury post-MI. Our findings break new ground by demonstrating that cardiomyocyte-specific deletion of *Bmal1* eliminates inherent circadian variation in myocardial injury and cardiac function impairment during both the acute phase and extended reperfusion periods. Our research primarily focuses on BMAL1 in cardiomyocytes, given its central role in cardiac dysfunction and post-MI heart failure. However, considering the prevalence of peripheral molecular clocks in numerous cell types within the heart^31^, BMAL1’s function in these cells may also contribute to the circadian modulation of myocardial injury. Studies have shown that endothelial BMAL1 governs diurnal variation in both thrombotic responses^83^ and the activation of plasminogen activator inhibitor-1 (PAI-1)^84^, a protein crucial for regulating the fibrinolytic system. Given the pivotal role of the dynamic balance between thrombus formation and fibrinolysis in increased MI risks and reduced efficacy of thrombolytic therapies during the morning hours, BMAL1’s involvement in this context is of significant importance. Furthermore, BMAL1’s role in circadian-dependent myocardial injury appears to be highly cell-specific. For instance, targeted deletion of *Bmal1* in smooth muscle cells disrupts myogenic reactivity, resulting in the suspension of myogenic tone at the circadian minimum^85^. This phenomenon leads to reduced total peripheral resistance and improved cardiac function post-MI^85^. Another study demonstrated that BMAL1 governs diurnal aging in neutrophils, and persistent aged neutrophils in the vasculature elevate thrombo-inflammation risks^86^. Thus, neutrophil-specific deletion of *Bmal1* results in defective aging, contributing to reduced infarction post-MI^86^. These findings, combined with our current research, underscore the diverse and highly cell-specific functions of BMAL1 in modulating circadian variations of myocardial injury.

While extensive research has focused on BMAL1’s interaction with its canonical partner, CLOCK, in the fine-tuning of clock-controlled genes, there remains a significant gap in our understanding of BMAL1’s partners in various pathological contexts, notably hypoxic conditions. In this regard, previous studies suggested that BMAL1 associates with HIF2A to form a transcriptionally active complex, as demonstrated in cellular systems through yeast two-hybrid and luciferase reporter assays^67,78^. However, the direct and stable interaction between BMAL1 and HIF2A had not been confirmed until a recent study involving plateau pika^79^. In this study, HIF2A, harboring a unique highland-adaptive mutation, was shown to directly bind to BMAL1, forming a stable complex capable of recognizing E-box DNA^79^. Notably, the majority of evidence supporting BMAL1/HIF2A interaction and its functionality relies on overexpressed proteins^67,78,79^. To accurately reflect true endogenous interactions, our work, for the first time, unveils a circadian-dependent interaction between endogenous BMAL1 and HIF2A in human cardiomyocytes under hypoxic conditions. Additionally, we demonstrate an enhanced co-localization of BMAL1 and HIF2A in the nuclei of ischemic mouse hearts, especially at ZT8. This revelation provides a deeper understanding of their synergistic activity and diurnal impact on circadian-dependent cardioprotection. To further gain a molecular-level understanding of heterodimerization of BMAL1 with HIF2A for DNA binding, we determined the cryo-EM structure of the BMAL1/HIF2A/DNA complex. This structure provides compelling structural evidence that supports the long-theorized formation of the BMAL1/HIF2A heterodimer and its intrinsic ability to bind to DNA. Future research can delve into potential therapeutic applications or interventions based on this structure, especially considering the significance of these molecules in circadian rhythms and the cellular hypoxia response.

HIF2A plays a crucial role in the body’s adaptive response to hypoxia^2,63–66^. Under normoxic conditions, HIF2A undergoes rapid degradation. In contrast, during hypoxic conditions, HIF2A is stabilized. Once stabilized, it dimerizes with HIF1B and initiates the transcription of target genes, which offer protective effects, notably during myocardial IRI^6,63,66^. Several studies have shown that both genetic and pharmacological strategies aimed at inhibiting HIF degradation consistently provide cardioprotection^23,66,87–91^. However, the specific stabilization pattern of HIF2A and its influence on the diurnal variation of myocardial injury have remained elusive. Our novel findings reveal that conditions like hypoxia and myocardial IRI induce circadian-dependent stabilization of HIF2A and its rhythmic binding to the promoter region of the target gene, *AREG*. We further demonstrate that similar to *Bmal1* ablation, cardiomyocyte-specific deletion of *Hif2a* eliminates the endogenous cardioprotection observed at ZT8 (when HIF2A stabilization is significantly enhanced in control mice) during both the acute phase and extended reperfusion periods following IRI. This discovery opens the door to pioneering therapeutic approaches. By selectively stabilizing or activating HIF2A at specific times of the day, we can maximize its inherent cardioprotective benefits during more vulnerable periods, potentially mitigating the side effects associated with prolonged HIF2A stabilization^23,92^.

Previous studies have identified *AREG* as a critical target gene of HIF2A during myocardial IRI, contributing to cardioprotection^66,68^. For instance, microarray analyses conducted on mouse models with genetic deletion of *Hif2a* have highlighted the pivotal role of HIF2A in the induction of *AREG*^66^. This is corroborated by increased HIF2A and AREG levels in myocardial tissues from patients diagnosed with ischemic heart disease^66^. A novel aspect of our research is the revelation of a diurnal pattern in *AREG* induction, observed both in hypoxic cardiomyocytes and in ischemic mouse hearts. Further, we decipher the core mechanism of this rhythmic induction: BMAL1 and HIF2A can interact and bind directly to a highly conserved motif (CAGGTG) within the *AREG* promoter during ambient hypoxia. Moreover, this interaction between BMAL1 and HIF2A is indispensable for their synergistic transactivation of *AREG*. Intriguingly, mice lacking *Areg* exhibit diminished daytime variations of myocardial injury post-MI, indicating a circadian-dependent cardioprotective role of AREG. Thus, our research illuminates a previously unexplored, endogenous regulatory mechanism that dictates circadian variation in myocardial injury via the BMAL1/HIF2A-*AREG* pathway. We further demonstrate that timed AREG administration, especially at its endogenous expression trough, presents an exciting avenue for enhancing cardioprotection. Therefore, our findings indicate the significance of considering the timing of drug administration to optimize treatment strategies, particularly when a strong correlation exists between the pharmacodynamic effect and the plasma or tissue levels of a drug that acts on a therapeutic target, which is itself under circadian regulation^30,68^. This is highly relevant considering that over half of the top-selling 100 drugs in the United States target genes with circadian rhythms and half-lives of less than 6 hours^93^. For instance, nighttime administration of hypertensive medications and aspirin improves their efficacy during the morning vulnerable period and reduces side effects^30,94,95^. Furthermore, in alignment with our findings that targeting circadian rhythm could reduce myocardial injury, other studies have shown that circadian rhythm disruption, such as in shift work, exacerbates myocardial infarction reperfusion injury^39^. Collectively, these findings highlight the potential of chronotherapy, involving either optimizing the timing of drug administration or directly enhancing circadian rhythms to maximize therapeutic efficiency and minimize adverse effects.

On top of these findings, our structural analysis of the BMAL1/HIF2A/DNA complex reveals the ability of BMAL1 to undergo significant conformational rearrangements when interacting with different partners in response to diverse regulatory conditions. This structural insight also lays the foundation for future targeted drug discovery. Taken together, the present study unravels a novel mechanism of the BMAL/HIF2A-AREG pathway in modulating circadian variation of myocardial injury. Leveraging this pathway using circadian-based treatment strategies significantly improves the efficacy of cardioprotection for treating MI.

## Methods

### Human subject research

#### Study design and participants:

We examined samples from a prospective study of myocardial injury in humans during cardiac surgery (clinicaltrials.gov: NCT00281164). The study population consisted of consecutive patients (aged ≥20 years) with aortic stenosis referred to our cardiovascular surgery department at Brigham and Women’s Hospital (MA, USA) for aortic valve replacement (with or without coronary artery bypass graft) between Jan 1, 2009, and Dec 31, 2014. Patients enrolled in a concurrent drug or device trial were excluded. This ongoing study involved 56 patients in the morning (samples collected between 8:00 am-12:00 pm, median time 10:32 am) and 17 patients who underwent the same procedure in the afternoon (samples collected between 3:00–9:00 pm, median time 5:15 pm). Patients whose surgery fell outside of these time periods were excluded from the analysis. The ethics committee of our institution approved the protocol, and written informed consent was obtained from all patients.

#### Procedures:

Patients underwent aortic valve replacement either in the morning or in the afternoon by the same senior surgeon. Anesthesia, cardiopulmonary bypass, cardioplegia, and surgical procedures were done according to standard guidelines. Anesthesia was induced with intravenous fentanyl or sufentanil and propofol (0.5–1.5 mg/kg) and maintained with isoflurane. Surgery was done using normothermic cardiopulmonary bypass and repeated antegrade and retrograde cold blood cardioplegia. Left ventricular biopsy samples from the morning and afternoon patient cohorts were obtained after approximately 80 minutes of aortic cross-clamping at the site of the routinely placed left ventricular vent.

### Animal care and use

Animal care was performed according to the guide for the care and use of laboratory animals of the National Institutes of Health. All experimental procedures were approved by the UTHealth Institutional Animal Care and Use Committee. The sample size was estimated based on published literature on previous murine models of myocardial ischemia and reperfusion injury^5^. All mice were housed in a standard 12 h light:12 h darkness (L/D) photoperiod at 22°C degrees with ad libitum access to a standard chow diet.

### Generation of transgenic mouse models

To generate cardiac myocyte-specific deletion, Myosin Cre+ (STOCK *A1cf*^Tg[Myh6–cre/Esr1*]1Jmk/^J, The Jackson Laboratory, RRID:IMSR_JAX:005650), *Hif1a*^*loxP/loxP*^ (B6.129-*Hif1a*^*tm3Rsjo*^/J, The Jackson Laboratory, RRID:IMSR_JAX:007561), *Hif2a*^*loxP/loxP*^ (STOCK *Epas1*^*tm1Mcs*^/J, The Jackson Laboratory, RRID:IMSR_JAX:008407), and *Bmal1*^*loxP/loxP*^ (B6.129S4(Cg)-*Bmal1*^*tm1Weit*^/J, The Jackson Laboratory, RRID:IMSR_JAX:007668) mice were purchased from The Jackson Laboratory (Bar Harbor, ME) and crossbred as previously described^5,66^. To address sex as a biological variable, both male and female mice, aged 8 to 16 weeks, underwent myocardial ischemia and reperfusion surgery. Our sex-specific analysis, which included assessments of infarct size, troponin levels, and cardiac function, revealed no significant differences between sexes^3,96^. For the induction of Cre-recombinase activity, mice received an i.p. injection of tamoxifen at a dosage of 1 mg/day for five consecutive days, as previously described^3,96,97^. A recovery period of seven days was allowed following the final tamoxifen dose before proceeding with further experimental procedures. In these experiments, Myosin Cre+ mice served as controls. Additionally, *Areg*^*−/−*^ mice (B6;129-*Areg*^*tm1Dle*^/Mmnc, MMRRC, MMRRC_011533-UNC)^98^ were purchased from Mutant Mouse Resources & Research Centers (Chapel Hill, NC). To circumvent lactation difficulties observed in younger females, *Areg*^*−/−*^ mice were bred using a heterozygous (*Areg*^/−^) strategy^66,68^. Control mice for these experiments were C57BL/6J (The Jackson Laboratory, 000664), chosen for their same genetic background as the *Areg*^*−/−*^ mice. Genotyping for all mouse strains was conducted by GeneTyper Inc. (NY, USA).

### Murine model of myocardial ischemia and reperfusion injury (IRI)

The mouse model used in the study was subjected to a period of entrainment for at least two weeks within circadian cabinets (Actimetrics, IL, USA) to establish a stable circadian rhythm^73^. Following this acclimatization period, the mice underwent an in-situ procedure for myocardial ischemia and reperfusion injury at different times of day (ZT2, ZT8, ZT14, or ZT20), as detailed in prior publications^3,5,96,97,99^. In brief, the mice were anesthetized using 1–3% isoflurane delivered by a Compact Small Animal Anesthesia Device (RWD, TX), followed by endotracheal intubation and ventilation using a VentStar Small Animal Ventilator (RWD, TX). Once anesthetized, they were placed in a supine position and received pre-incisional analgesia with sustained-release buprenorphine (0.1 mg/kg s.c.; ZooPharm, AZ). Throughout the procedure, their body temperature was consistently maintained at 37°C with a ThermoStar Homeothermic Blanket equipped with rectal feedback control (RWD, TX). The surgical procedure was meticulously performed using a research stereomicroscope system, SZX10 (Olympus, USA). We temporarily ligated the proximal left anterior descending coronary artery approximately 2 mm from its emergence beneath the left atrium, utilizing Surgipro II 7–0 monofilament polypropylene sutures (Covidien). The success of this ligation was confirmed by observing blanching or a pale discoloration of the anterior wall of the heart, a reduction in wall movement, and the presence of ST-segment elevation on an electrocardiogram (ECG). Following 45 minutes of induced ischemia, reperfusion was initiated to restore blood flow. The effectiveness of reperfusion was verified both by the resolution of ST-segment elevation on the ECG and through direct visual inspection, where a return of color and movement in the previously affected area of the heart was noted. Post-surgery, the mice were carefully transferred back to the circadian cabinets for recovery and continued observation. Any mice that did not survive within the first 48 hours post-MI were considered to have experienced technical complications and were consequently excluded from further analysis.

### Timed administration of AREG and NOB in mouse models

#### Timed administration of AREG:

In the pre-ischemia AREG treatment experiments, mouse recombinant AREG protein (R&D Systems, MN) was prepared in 0.9% NaCl solution and a dosage of 10 μg AREG was administered intravenously 30 minutes prior to surgery. This administration was carried out using a syringe pump connected to an indwelling catheter placed in the carotid artery. To simulate the clinical scenario where treatment is initiated after the onset of MI, AREG at the same dosage (10 μg) was administered at the start of the reperfusion phase. Furthermore, to assess the long-term effects of timed AREG treatment on cardiac function, a post-injury treatment regimen was implemented. In this protocol, AREG (10 μg) was administered via i.p. injection at either ZT8 or ZT20 daily for the first three days following myocardial IRI. For control purposes, an equivalent volume of 0.9% NaCl solution was administered as a vehicle.

#### Administration of NOB:

In the NOB treatment experiments, Mice were administered either DMSO (as a vehicle control) or NOB at a dose of 200 mg/kg body weight. The administration was carried out via i.p. injection and repeated on an every-other-day basis. This treatment protocol was followed for two weeks prior to the surgical procedure and was specifically timed within the ZT14-ZT20 time window. To evaluate the long-term effects of NOB on cardiac function and remodeling, the same dosing regimen was continued following IRI. The chosen dosing regimen was based on several considerations: Firstly, the dosage range was consistent with those used in previous studies (100–200 mg/kg/day)^73,74^, and it was aligned with the active phase of the mice. Secondly, daily dosing was intentionally avoided to prevent the entrainment of the experimental mice to the dosing schedule as an artificial zeitgeber. Thirdly, previous single-dose pharmacokinetic (PK) assays demonstrated a favorable PK profile for NOB, with significant exposure detected in the serum, brain, and liver^73^. Considering that NOB levels were typically undetectable within 8–24 hours post-administration^73,100^, we opted for an every-other-day dosing strategy to prevent incomplete clearance over the 4-week experimental period.

### Mouse RNA-sequencing (RNA-seq) and bioinformatic analysis

The AAR of the heart was harvested following 2h of reperfusion from the C57BL/6J mice subjected to myocardial IRI at either ZT8 or ZT20. Total RNA was extracted using the RNeasy Mini kit (Qiagen) and employed to construct RNA-Seq libraries, which were then sequenced using the Illumina 1.9 platform (75 bp pair-end). Alignment of RNA sequencing tags was restricted to those mapping to the same DNA strand as annotated in the GRCm38 reference genome, utilizing the STAR software. Quantification of transcripts was conducted by calculating the fragments per kilobase of transcript per million mapped reads (FPKM) values, along with transcript counts. In the preprocessing step of mRNA analysis, any gene identified as non-expressed, defined as having an FPKM expression level less than 1 in more than 80% of samples, was excluded. The normalized expression profiles of these mRNAs were then subjected to PCA for quality control and to evaluate sample similarity. Differential expression analysis was executed using the DESeq2^101^ pipeline, with DEGs being identified based on a threshold of 1.5-fold change and an adjusted p-value below 0.05, as determined by the Benjamini-Hochberg method^102^.

The functional enrichment analysis of the identified DEGs was conducted utilizing the GO and KEGG pathway databases through the WebGestalt tool^103^ (http://www.webgestalt.org/, version 2019). For rigorous validation, only pathways and GO terms with an adjusted p-value of less than 0.05 were included. In constructing the gene regulation network, we utilized known TF-mRNA interactions from the TRRUST_v2^104^ and Chipbase_v2^105^ databases. Additionally, the mouse protein-protein interaction (PPI) network was integrated from the STRING database^106^, with a focus on interactions having a combined score above 900. Considering the limited sample size at each time point, we merged six samples from both conditions to calculate Spearman’s Correlation Coefficient (SCC), ensuring robust and meaningful analysis. In finalizing the gene regulation network, we merged the TF-mRNA regulation network with the PPI data, specifically targeting interactions where both genes were differentially expressed. We also selectively incorporated edges with high SCC into our network, tailored to align with the specific conditions of our experimental design.

### Human RNA-seq and bioinformatic analysis

For human RNA-seq and subsequent bioinformatic analysis, we began by excluding outlier samples that failed to cluster appropriately, resulting in a total of 73 samples for analysis, including 56 morning and 17 afternoon samples derived from various sequencing protocols. Quality control of raw sequencing reads identified adaptor sequences from the Illumina Nextera platform in some samples, which were subsequently trimmed using Cutadapt^107^. kallisto (v0.46.1)^108^ was used to quantify transcript level expression by mapping to a transcript index built from GENCODE human transcript V44^109^. We then employed tximport to convert these transcript-level quantifications to gene read counts^110^. Differential expression analysis was performed with DESeq2 (v1.34.0)^101^, with particular attention paid to sequencing batch to control for batch effects. Genes displaying a log2 fold change greater than 0.5 and a p-value less than 0.01 were deemed significant. For enrichment analysis, we used the GO annotation version 1.1 for humans, employing all genes annotated by at least one GO term^111^ as the background in Fisher’s exact test to identify over-represented biological processes. Additionally, the R package KEGGREST was employed to extract KEGG pathway annotations and Cytoscape 3.10.0 was utilized to construct network plots for the identified KEGG pathways.

### Microarray data re-analysis

The microarray assay for gene expression transcript levels of post-ischemic myocardium from Myosin Cre+ or *Hif2a*^*loxP*/*loxP*^ *Myosin Cre+* mice was obtained from GEO with accession number GSE67308^66^. Differential expression was carried out by GEO2R with the selection of applying limma precision weights while other options remained default^112^.

### Assessment of infarct size in the acute phase.

The assessment of myocardial infarct size was conducted by determining the percentage of infarcted myocardium within AAR, utilizing a previously established method^3,5,66,96,97^. Following 2 hours of reperfusion, the hearts were flushed with PBS and then subjected to permanent occlusion of the left coronary artery. 1 ml of 1% Evans blue dye (Sigma-Aldrich, E2129) was then infused through the carotid artery catheter. Post infusion, the hearts were excised and sectioned into 1 mm slices using a microtome (Roboz, SA-4130). These heart sections were subsequently double-stained with 1% 2,3,5-Triphenyltetrazolium Chloride (TTC; Sigma-Aldrich, T8877) for 10 minutes at 37°C and then fixed in 10% formalin overnight. The double-stained heart slices were photographed, and ImageJ software (NIH) was employed to calculate the infarct size.

### Cardiac troponin I ELISA

Serum samples were collected from mice subjected to myocardial IRI at various ZTs through the inferior vena cava following 2h of reperfusion. The measurement of serum troponin I, a highly sensitive biomarker for cardiac injury, was conducted using the mouse cardiac troponin-I SPARCL^™^ kit (Life Diagnostics, CTNI-SP-1), as we have done previously^3,5,66,96,97^.

### Speckle-tracking echocardiography for assessing mouse cardiac function

#### Ultrasound imaging:

Cardiac function and structure were evaluated using transthoracic echocardiography with a Vevo3100 Ultrasound system (VisualSonics, Toronto, Canada). Mice were mildly sedated using 0.5–1.0% isoflurane and placed on a heated platform equipped with ECG monitoring. Validation criteria included stable and continuous endocardial tracking throughout the cardiac cycles, ensuring a heart rate of around 450–550 beats per minute and a frame rate above 250 frames per second. Two-dimensional gray-scale echocardiographic images were captured from both parasternal long-axis and short-axis views. Specifically, the longitudinal strain was indicative of myocardial shortening at the endocardium level, while the radial strain reflected shortening at the mesocardium. All image acquisitions and subsequent offline measurements were conducted by a single investigator who was blinded to the grouping of the animals.

#### Speckle-based deformation mapping and analysis:

For strain analysis, we meticulously chose appropriate B-mode loops, ensuring clear visualization of the endocardial border and absence of image artifacts. Three consecutive cardiac cycles with distinct ECG recordings were chosen for detailed analysis. Semi-automated tracing of both the endocardial and epicardial borders was carried out, with adjustments made as required to ensure optimal tracking quality throughout each cine loop. These tracked images were then processed frame by frame to conduct strain measurements^42–44^. The resulting strain values were averaged across these cardiac cycles, providing comprehensive data on the LV systolic function (EF, FS, and GLS), LV size (EDV and ESV), and LV mass (EDLVM and ESLVM).

In the analysis of regional and global cardiac dynamics, including contractility and synchronicity, the LV endocardium was mapped using 48 sampling points^43,44^. This approach divided the chamber into six segments for an in-depth examination in the long-axis view: basal anterior, mid anterior, apical anterior, basal posterior, mid posterior, and apical posterior segments^44^. In the myocardial IRI models, the mid anterior, apical anterior, and apical posterior wall segments were delineated as the “infarct” region, with the remaining segments classified as the ‘non-infarct’ region^43^. Peak systolic strain, representing the percentage change in length during myocardial contraction and relaxation, was calculated using the formula: ε = (L1 – L0)/L0, where L0 is the original length, and L1 is the final length^113^. This calculation was performed for each segment to assess peak systolic strain (%) and time-to-peak systolic strain (ms). Abnormal ventricular contractility patterns were assessed based on systolic strain magnitude (segment peak strain) and timing (peak of shortening). Specifically, dyssynchrony was characterized by a pattern of reduced systolic strain magnitude, early opposite deflection, and delayed time-to-peak systole. Akinesis was identified as minimal or no contractility, with peak systolic strain ranging between −5 % and 5%. Dyskinesis was described as ventricular systolic motion occurring in the opposite direction to normal contraction. Additionally, intra-ventricular disparity was quantified by intra-ventricular delay in time-to-peak strain and standard deviation of time-to-peak strain across segments^43,114^.

### Chromatin Immunoprecipitation followed by quantitative Polymerase Chain Reaction (ChIP-qPCR)

HCMs purchased from ScienCell Research Laboratories (ScienCell, 6200) were isolated from human heart^115^. HCMs were synchronized by dexamethasone (200 nM) for 1h and then exposed to normoxia or hypoxia (1% oxygen) for 4h at CT20 and CT32 in Fig. 3q. HEK293 cells were transfected with pcDNA3 *mBmal1* (a gift from Aziz Sancar, Addgene, 31367)^72^ and treated with normoxia or hypoxia (1% oxygen) for 4h in Fig. 3s. ChIP-qPCR was conducted with the SimpleChIP^®^ enzymatic chromatin IP kit (Cell Signaling Technology, 9002). In brief, cells were fixed with 1% formaldehyde, quenched with 125 mM glycine, washed, and sonicated to shear the DNA to an average length of 200–500 bp. Lysates were then incubated overnight at 4°C with ChIP grade anti-HIF2A antibody (Novus Biologicals, NB100–122) and anti-BMAL1 antibody (Cell Signaling Technology, 14020), or IgG as a negative control. After washing, the antibody-protein-DNA complexes were eluted from the beads. The cross-links were then reversed through overnight incubation at 65°C, followed by DNA purification. For the qPCR analysis, specific primer pairs were designed to amplify the predicted common binding sequence (CAGGTG) on the human *AREG* promoter region. The enrichment was quantitatively compared against input controls and IgG negative controls to confirm the specificity of the ChIP-qPCR assay.

### Co-immunoprecipitation (Co-IP)

HEK293 cell lines (Fig. 2b and Extended Data Fig. 6b) overexpressing the pcDNA3 *mBmal1* (a gift from Aziz Sancar, Addgene, 31367)^72^ were exposed to either normoxia or hypoxia (1% oxygen) for 4 hours. In Fig. 3r, HCMs were initially synchronized using dexamethasone (200 nM) for 1h and then exposed to hypoxia (1% oxygen) for 4h at CT20 and CT32. After exposure, the cytoplasmic and nuclear fractions were separately extracted using the NE-PER^™^ Nuclear and Cytoplasmic Extraction Kit (ThermoFisher Scientific, 78835), as per the manufacturer’s instructions. The cell lysates were centrifuged and pre-cleared with protein A/G beads (ThermoFisher Scientific, 53133) for 1 hour with rotation. For the IP, the pre-cleared lysates were incubated overnight at 4°C with specific antibodies: mouse anti-FLAG (Sigma-Aldrich, F1804) or rabbit anti-HIF2A (Novus Biologicals, NB100–122) antibodies. Mouse IgG (Cell Signaling Technology, 5415) and rabbit IgG (Abcam, ab172730) were used as isotype controls for the respective antibodies. Following the overnight incubation, 40 μl of Protein A/G beads were added to each sample, and the incubation was continued for an additional 4 hours at 4°C. The proteins were then eluted using an SDS sample buffer and subjected to heating at 95°C for 5 minutes with vigorous shaking. The eluted proteins were subsequently stored at −80°C for further analysis. In Fig. 2c, HCMs were subjected to hypoxic conditions (1% oxygen) for various durations: 0, 0.5, 1, 2, 4, or 8 hours. Following each exposure period, HCMs from a single 10 cm dish were lysed using 330 μl of HEPES EB buffer containing 20 mM HEPES pH 7.4, 100 mM NaCl, 1 mM EDTA, 0.1% Triton X-100, 5% glycerol, and a cocktail of protease, phosphatase, and RNase inhibitors. Co-IP was then performed following the previously described protocol.

### Proximity ligation assay (PLA)

Confluent HCMs were subjected to hypoxic conditions (1% oxygen) for various durations (0, 1, 4, or 8 hours). Post-exposure, the cells were fixed using 4% paraformaldehyde (Millipore Sigma, P6148), permeabilized with 0.1% Triton X-100 (Amresco, M143), and subjected to a blocking step to reduce non-specific binding. Rabbit anti-BMAL1 (Abcam, ab3350), mouse anti-HIF2A (Novus Biologicals, NB100–132), and mouse anti-HIF1A (Novus Biologicals, NB100–105) were then applied. For PLA, pairs of primary antibodies raised in different species were used as mentioned. After primary antibody incubation, cells were treated with Duolink^®^ In Situ PLA^®^ Probes (Millipore Sigma, DUO92002 and DUO92004) conjugated to these antibodies. These probes, when in close proximity of less than 40 nm, facilitated the ligation of adjacent oligonucleotides attached to them. Subsequently, the ligated oligonucleotides were amplified and detected using fluorescently labeled probes. The resulting fluorescence signals, which are indicative of protein-protein interactions, were visualized using a Nikon Eclipse Ti2 confocal microscope (Nikon, USA) and analyzed with NIS Element AR software (Nikon). To confirm the specificity of the observed interactions, control experiments were performed using only the BMAL1 antibody.

### Plasmid construction and site-directed mutagenesis

To overexpress proteins in *Escherichia coli*, the DNA sequences encoding the N-terminal portions of mouse HIF2A (residues 3–361) and mouse HIF1A (residues 13–357), with a 6×His tag at their N-terminus, were inserted into a pET15b vector, respectively. The DNA encoding the N-terminal region of mouse BMAL1 protein (residues 68–488), with a C-terminal Flag tag, was ligated into a pET24b vector.

For GST pull-down, the DNA encoding residues 3–361 of mouse HIF2A was inserted into pGEX-6P-1 to generate GST-HIF2A for expression. Six BMAL1 single-site mutants (L95E, L115E, L150E, F248D, R343A and F423D) and three double-site mutants (L95E/L115E, L150E/F248D, and R343A/F423D) were generated using Quick-change Site-Directed Mutagenesis. The DNA sequences of all mutants were verified by DNA sequencing.

### Protein expression and purification

The recombinant plasmids were individually transformed into *E. coli* BL21 Rosetta (DE3) or co-transformed together to express either individual proteins or the BMAL1/HIF2A complex. Expression of the proteins or BMAL1/HIF2A complex was carried out at 18 °C by induction with 0.1 mM IPTG (isopropyl-β-d-thiogalactoside) for 16 hours. The BMAL1/HIF2A heterodimer (including bHLH, PAS-A, and PAS-B domains) was first purified through a Ni-NTA affinity column (ThermoFisher Scientific, 88222) followed by Heparin chromatography. The heterodimer eluted from the Heparin column was further analyzed by size exclusion chromatography using a Superdex 200 Increase 10/300 GL column. The peak fractions were assessed using SDS-PAGE and Coomassie blue staining. Subsequently, they were combined and concentrated. The concentrated protein was then used for cryo-EM sample preparation or stored at −80 °C for further use. His-HIF2A and His-HIF1A were purified individually using Ni-NTA affinity columns, followed by dialysis to eliminate imidazole. The proteins were concentrated and stored at −80 °C for further use.

### Pull-down assay

To confirm the interaction between BMAL1 and HIF2A or HIF1A in Fig. 2g, BMAL1-expressing cells were lysed using binding buffer [20 mM HEPES (pH 7.4), 10% glycerol, 0.01% Triton X-100, 300 mM NaCl, 5 mM MgCl_2_, 1 mM DTT, and protease inhibitors]. Following centrifugation, the supernatants were incubated with anti-Flag M2 beads (Millipore Sigma, A2220) for 40 min at 4 °C. Subsequently, the beads were washed three times using the binding buffer, followed by the addition of purified His-HIF2A or His-HIF1A proteins. Following 40 min incubation at 4 °C, the beads were washed five times using the binding buffer. The proteins bound to beads were then eluted using Flag peptide (0.3 mg/mL) and further analyzed by SDS-PAGE and western blotting using anti-Flag (Sigma-Aldrich, F1804) and anti-His (ThermoFisher Scientific, MA1–21315) antibodies.

To compare the interactions between HIF2A and wild-type BMAL1 or BMAL1 mutants in Fig. 6e, the cell pellets of GST-HIF2A and GST (as control) were lysed by sonication in binding buffer [20 mM HEPES (pH 7.4), 10% glycerol, 0.05% Triton X-100, 300 mM NaCl, 5 mM MgCl2, 1 mM DTT, and protease inhibitors]. After centrifugation, the supernatants were incubated with Glutathione magnetic agarose beads (GE Healthcare, 17-0756-01) for 30 min at 4 °C. The beads were subsequently washed three times using the binding buffer, followed by the addition of Flag-tagged wild-type or mutated BMAL1. After 40 min incubation at 4 °C, the beads were washed five times using the binding buffer. The proteins bound to beads were eluted by heating with SDS-PAGE loading dye at 95 °C. The eluted samples were further analyzed by SDS-PAGE and western blotting using anti-GST (Genscript, A00865) and anti-Flag (Sigma-Aldrich, F1804) antibodies.

### Electrophoretic mobility shift assay (EMSA)

5 fmol of 22-bp biotin-labeled dsDNA fragment containing a HRE consensus sequence was incubated with 1, 2, 3, 4, and 6 pmol of purified BMAL1/HIF2A heterodimer in a total volume of 10 μl in 10 mM MES (pH 6.0), 50 mM KCl, 1 mM DTT, 2.5% glycerol, 0.05% NP-40 and 5 mM MgCl_2_. After incubation at room temperature for 20 min, the binding reactions were directly loaded onto a native 4–20 % polyacrylamide gel and electrophoresed in 0.5×TBE buffer. DNA on the gel was transferred onto a positively charged nylon membrane and detected using an HRP-conjugated streptavidin with chemiluminescent substrates.

### Surface plasma resonance (SPR)

The interaction analysis of the BMAL1/HIF2A heterodimer with 22-bp DNA duplexes (HRE or E-box) was performed using OpenSPR (Nicoya Life Sciences Inc). A 5’-end biotin-labeled dsDNA fragment (2 μM) containing either a HRE or E-box consensus sequence was immobilized in channel 2 of the biotin-streptavidin sensor, while channel 1 remained unmodified and served as a control. The BMAL1/HIF2A heterodimer at various concentrations (from 5 to 50 nM) was injected and flowed slowly at a rate of 20 μl/min over the sensor chip for 5 minutes in a running buffer (20 mM MES pH 5.5, 300 mM NaCl, 0.05% Tween 20, 0.02% BSA). Following the injection, a 10-minute dissociation phase was collected. The resulting data were analyzed using Trace Drawer Kinetic Data Analysis software, employing a one-to-one model, which assumes one monovalent ligand binding to one target.

### Transactivation Assay

To assess the transcriptional activation activity of HIF2A and BMAL1 on the promoter of human *AREG*, the 3×300 bp sequences (−300/0) from the human *BMAL1* promoter harboring the common binding sequence (CAGGTG) were PCR amplified and cloned into the pEZX-PG04-promoter luciferase vector to generate GLuc-*hAREG*. HEK293 cells were co-transfected with several constructs: pcDNA3 *mHif2a*-P405A/P530V/N851A (oxygen-regulation insensitive, a gift from Celeste Simon, Addgene, 44027)^71^, pcDNA3 *mBmal1* (a gift from Aziz Sancar, Addgene, 31367)^72^, *hAREG-gluc*, and SEAP vectors (GeneCopoeia, LF031). Transfection was performed using Lipofectamine 3000 reagent (Invitrogen). After 48 hours, the culture medium was harvested, and the transcriptional activity was quantified using the Secrete-pair Dual Luminescence Assay Kit (GeneCopoeia, LF031) on a luminometer.

### Immunofluorescence staining and TUNEL assay

Harvested heart tissues were fixed in 10% neutral buffered formalin and then dehydrated through a graded alcohol series. Following dehydration, the tissues were cleared in xylene and embedded in paraffin blocks. Sections of 5–6 μm thickness were prepared and underwent antigen retrieval. These sections were then blocked with diluted donkey serum, followed by overnight incubation with primary antibodies at 4 °C. In the immunofluorescence staining process, a range of primary antibodies was employed to specifically target and identify various proteins within the tissue sections. These primary antibodies included: AREG (Santa Cruz, sc-133234, 1:50), HIF2A (Novus Biologicals, NB100–122, 1:200; Novus Biologicals, NB100–132, 1:200), BMAL1 (Abcam, ab3350, 1:200), α-sarcomeric (Abcam, ab137346, 1:200), vimentin (Abcam, 45939, 1:200), and α-smooth muscle actin (Cell Signaling Technology, 19245, 1:200) antibodies. For antigen visualization, sections were then incubated with Alexa Fluorescence-conjugated secondary antibodies (Invitrogen, Carlsbad, CA, USA). Additionally, TUNEL staining was conducted using in situ Click-iT^™^ Plus TUNEL assay kits (ThermoFisher Scientific, C10617) according to the manufacturer’s protocol to detect apoptotic cells as previously described^116^. Samples were counterstained with DAPI (1μg/mL, Invitrogen, D3571) and mounted using SlowFade Gold Antifade reagent (Invitrogen, S36936). The stained sections were then imaged with Nikon Eclipse Ti2 confocal microscopy (Nikon, USA) and analyzed using ImageJ software (NIH).

### Real-time quantitative PCR (qPCR)

Total RNA was extracted from both tissue samples and isolated cells using the RNeasy Mini Kit (Qiagen, 74106) in accordance with the manufacturer’s guidelines. This procedure is consistent with our established protocols as detailed in previous publications^3,66,96^. After the extraction, complementary DNA (cDNA) was synthesized from the extracted RNA using the High-Capacity cDNA Reverse Transcription Kit (Thermo Fisher Scientific, 4368814). Quantitative real-time PCR analyses were then carried out using SYBR Green PCR Master Mix (Qiagen, 204145). The reactions were performed on the Bio-Rad CFX384 Touch Real-Time PCR Detection System. For the analysis of gene expression levels, we applied the comparative Ct (ΔΔCt) method. The final data were presented as mean expression ratios relative to β-actin, allowing for the comparison of gene expression across different samples.

### Western blot

Mouse tissues and isolated cells were prepared for Western blot analysis by lysing in RIPA lysis buffer (Thermo Fisher Scientific, 89900), supplemented with both protease (Thermo Fisher Scientific, 78425) and phosphatase inhibitor cocktails (Thermo Fisher Scientific, 78420). Protein concentrations were quantified, and 10–20 μg of total protein were separated on 4%–12% SDS-PAGE gels (Bio-Rad Laboratories). Following electrophoresis, proteins were transferred to membranes for immunoblotting. The membranes were probed with primary antibodies: anti-HIF1A (Novus, NB100–105), anti-HIF2A (Novus, NB100–122), anti-HIF1B (Cell Signaling Technology, 5537), anti-BMAL1 (Cell Signaling Technology, 14020), anti-CLOCK (Cell Signaling Technology, 5157), anti-RORα (Abcam, ab60134), anti-AREG (Santa Cruz, sc-74501), anti-caspase-3 (Cell Signaling Technology, 9662), anti-cleaved caspase-3 (Cell Signaling Technology, 9661), anti-Bax (Cell Signaling Technology, 2772), anti-FLAG (Sigma, F1804), anti-Lamin B1 (Cell Signaling Technology, 12586), anti-TBP (Cell Signaling Technology, 8515), anti-α-tubulin (Cell Signaling Technology, 2144), and anti-β-actin (Cell Signaling Technology, 4967). Following incubation with secondary antibodies, the membranes were developed using appropriate substrates. Signal intensity was detected and quantified using ImageJ software (NIH). The results were normalized to the appropriate internal control, and data were expressed as relative fold changes in protein levels.

### Cryo-EM sample preparation

The purified BMAL1/HIF2A heterodimer was mixed with a 2-fold molar excess of the HRE dsDNA, followed by incubation on ice for 20 min. The mixture was then applied onto a Superdex 200 Increase 10/300 GL column. The eluate fractions from the column were analyzed by SDS-PAGE and stained with Coomassie blue. Peak fractions corresponding to the BMAL1/HIF2A/DNA complex were combined and concentrated to 0.5 mg/mL for preparing cryo-EM specimens. In brief, 3 ul of the purified complex was applied onto freshly glow-discharged R 2/1 holey carbon 300 mesh copper grids (C-flat). The grids were then vitrified in liquid ethane using an FEI Vitrobot (Mark IV).

### Cryo-EM data collection and processing

Single-particle cryo-EM images were collected using an FEI Titan Krios (300 KV) electron microscope with a Gatan GIF Quantum K2 direct electron detector. The images were automatically acquired using EPU (Thermofisher) at underfocus values ranging from 1.0 to 2.5 μm with a pixel size of 0.85 Å/pixel. Each image was exposed for 9 s with a total dose of ~75 electrons per Å^2^, which was fractionated into 45 frames. The movie frames were aligned using MotionCor2^117^, resulting in a total of 9,013 micrographs. Gctf^118^ was used to estimate the parameters of contrast transfer function (CTF) for each image. Cryolo^119^ was used for particle picking, yielding a total of 375,167 particles. Multiple rounds of 2D classification were performed in RELION (v-3.1)^120^/Cryosparc(v-2.5)^121^ to eliminate particles without features, resulting in a stack of 193,871 particles. These were then used to generate a 3D *ab initial* model using Cryosparc. Multiple rounds of 3D classification were carried out in RELION, resulting in 83,335 particles, which were used for subsequent 3D refinement (4.5 Å resolution). CTF refinement and Bayesian polishing were carried out to generate 83,335 shiny particles, which were then subjected to 3D classification. Final 41,991 shiny particles were used for 3D refinement, resulting in a 3.7 Å reconstruction. The resolutions of final 3D maps were estimated using gold standard Fourier Shell correlation (FSC) curves with 0.143 criteria^122^, and local resolutions were calculated using RELION (Extended Data Fig. 17c–e).

### Model building and refinement

To build an atomic model of the BMAL1/HIF2A/DNA complex, crystal structures of HIF2A [PDB: 4ZP4] and BMAL1 [PDB: 4F3L], as well as a 22-bp B-form DNA duplex, were fitted into the cryo-EM density map of BMAL1/HIF2A/DNA by rigid-body fitting in Chimera^123^. This model was then manually built and adjusted in Coot^124^ and refined using real-space refinement in Phenix^125^ (Extended Data Table 3). In the final BMAL1/HIF2A/DNA atomic model, amino acids for HIF2A (3–9, 155–160, 204–218, and 359–361) and BMAL1 (68–73, 126–142, 210–241, 255–279, 291–311, 322–337, and 443–488) were not built because of missing or poor densities. All molecular graphic figures were generated by Chimera, ChimeraX^126^ and PyMOL^127^.

### Quantification and Statistical Analysis

Unless otherwise specified, all results are presented as mean ± SEM, with the precise number of biological replicates (n) detailed in the figure legends. All data were plotted from independent biological replicates. The Shapiro-Wilk normality test was utilized to assess normal distribution. The equality of variances for unpaired *t*-tests was determined using the F-test. Statistical analysis was with GraphPad Prism 10.0 software, employing Student’s t-test (Two-tailed), Welch’s t tests (for unequal variances), Mann-Whitney U test (when normality assumptions were not met), or one-way ANOVA with Bonferroni’s multiple comparisons analysis, depending on the data set. Outliers were identified using the ROUT method (Q = 1%) in GraphPad Prism. For ChIP-qPCR data analysis in Fig. 3s, values identified as outliers were excluded from the statistical analysis. Cardiac function assessment in *Bmal1*^*loxP/loxP*^ Myosin Cre+, *Hif2a*^*loxP/loxP*^ Myosin Cre+, and *Hif1a*^*loxP/loxP*^ Myosin Cre+ mice was compared against a shared Myosin Cre+ control group, optimizing research efficiency by minimizing animal usage and ensuring a consistent baseline for accurate comparative analysis.

## Extended Data

**Extended Data Table 1.** Patient demographics, preoperative and intraoperative characteristics in human RNA-seq study.

	Morning surgery (n = 56)	Afternoon surgery (n = 17)	p-value
**Demographics**			
Sample collected time (median)	10:32 am	5:15 pm	
Age, years	70.57 (13.47)	72.23 (11.32)	0.85
Male sex	32 (57.14%)	13 (76.47%)	0.25
**Risk factors and comorbidities**			
Diabetes mellitus	20 (35.71%)	5 (29.41%)	0.77
Hypertension	44 (78.57%)	9 (52.94%)	0.06
Renal disease	3 (5.36%)	0 (0.00%)	0.99
Pulmonary disease	12 (22.64%)	3 (17.65%)	0.99
Smoking	31 (55.36%)	5 (29.41%)	0.10
**Cardiac status**			
New York Heart Association class	2.38 (0.63)	2.59 (0.51)	0.30
Left ventricular ejection fraction, %	60.59 (7.82)	58.69 (13.59)	0.78
Chronic atrial fibrillation	7 (12.50%)	3 (17.65%)	0.69
paroxysmal atrial fibrillation	3 (5.36%)	0 (0.00%)	0.99
Previous MI	4 (7.41%)	1 (5.88%)	0.99
**Preoperative medication**			
Aspirin	33 (64.71%)	11 (78.57%)	0.52
Beta-blockers	30 (58.82%)	7 (50.00%)	0.56
Angiotensin-converting enzyme inhibitors	23 (45.10%)	6 (42.86%)	0.99
Statins	40 (78.43%)	11 (78.57%)	0.99
Diuretics	22 (41.51%)	6 (42.86%)	0.99
**Surgery characteristics**			
Cardiopulmonary bypass duration, min	100.40 (35.33)	102.20 (42.33)	0.92
Aortic cross-clamp duration, min	78.36 (24.33)	82.12 (36.36)	0.90
Concomitant coronary artery bypass graft	23 (41.07%)	9 (52.94%)	0.42

Data are mean (SD) or n (%).

**Extended Data Table 2.** PCR-based array analysis of the top 20 putative HIF2A target genes.

	Gene Symbol	Fold change (ZT8 vs.ZT20)
1	*Areg*	5.336429281
2	*Camp*	4.776067317
4	*Ppp2r3a*	2.050049876
3	*Ereg*	1.385175458
5	*Fam84a*	1.089538281
6	*Ifit3*	1.008826154
7	*Olr1*	0.961149995
8	*Trim6*	0.906638861
9	*Cmpk2*	0.848709008
10	*Serpina3n*	0.796317083
11	*Ngp*	0.726639657
12	*4930402F11Rik*	0.699162661
13	*Gbp5*	0.680533104
14	*Otud1*	0.601659987
15	*Cd274*	0.541292184
16	*Ifit2*	0.516523501
17	*Csf3*	0.423441291
18	*Cxcl10*	0.423184196
19	*Rsad2*	0.397828297
20	*Cxcl11*	0.32245024

**Extended Data Table 3.** Cryo-EM data collection, processing, and refinement.

**Data collection**	
Microscope	Titan Krios
Voltage (KV)	300
Camera	GIF Quantum K2
Defocus range (μm)	1.0 to 2.5
Pixel size (Å)	0.85
Movies	9,013
Frames per movie	45
Exposure time per frame (ms)	200
Magnification	165,000x
Dose rate (e^−^/pixel/sec)	6
Total dose per movie (e^−^/Å^2^)	75
**Data processing**	
Map	BMAL1/HIF2A/DNA
Particles	41,991
Symmetry imposed	C1
Map Resolution (Å)	3.7
Map sharpening B factor (Å^2^)	−80
**Model building and refinement**	
Model	BMAL1/HIF2A/DNA
Initial model used (PDB)	4ZP4; 4F3L
Protein residues	578
Nucleotide	44
Metals	0
Other atoms	0
R.m.s deviations	
Bond angles (°)	1.416
Bond lengths (Å)	0.007
Ramachandran plot	
Favored (%)	94.18
Outlier (%)	0.00
Validation	
Clash score	6.31
MolProbity score	1.74

**Extended Data Table 4.** Key resources table

REAGENT or RESOURCE	SOURCE	IDENTIFIER
**Antibodies**		
Rabbit polyclonal anti-HIF1A	Novus Biologicals	Cat # NB100–479 RRID:AB_10000633
Mouse monoclonal anti-HIF1A	Novus Biologicals	Cat # NB100–105 RRID:AB_10001154
Rabbit polyclonal anti-HIF2A	Novus Biologicals	Cat # NB100–122 RRID:AB_10002593
Mouse monoclonal anti-HIF2A	Novus Biologicals	Cat # NB100–132 RRID:AB_10000898
Rabbit monoclonal anti-HIF1B	Cell Signaling Technology	Cat # 5537 RRID:AB_10694232
Rabbit monoclonal anti-BMAL1	Cell Signaling Technology	Cat # 14020 RRID:AB_2728705
Rabbit polyclonal anti-BMAL1	Abcam	Cat # ab3350 RRID:AB_303729
Rabbit monoclonal anti-CLOCK	Cell Signaling Technology	Cat # 5157 RRID:AB_10695411
Rabbit polyclonal anti-RORα	Abcam	Cat # ab60134 RRID:AB_945289
Mouse monoclonal anti-AREG	Santa Cruz Biotechnology	Cat # sc-74501 RRID:AB_1118939
Rabbit polyclonal anti-caspase-3	Cell Signaling Technology	Cat # 9662 RRID:AB_331439
Rabbit polyclonal anti-cleaved caspase-3	Cell Signaling Technology	Cat # 9661 RRID:AB_2341188
Rabbit polyclonal anti-Bax	Cell Signaling Technology	Cat # 2772 RRID:AB_10695870
Mouse monoclonal anti-FLAG	Sigma-Aldrich	Cat # F1804 RRID:AB_262044
Rabbit monoclonal anti-Lamin B1	Cell Signaling Technology	Cat # 12586 RRID:AB_2650517
Rabbit polyclonal anti-TBP	Cell Signaling Technology	Cat # 8515 RRID:AB_10949159
Rabbit polyclonal anti-α-tubulin	Cell Signaling Technology	Cat # 2144 RRID:AB_2210548
Rabbit polyclonal anti-β-actin	Cell Signaling Technology	Cat # 4967 RRID:AB_330288
Rabbit monoclonal IgG	Abcam	Cat # ab172730 RRID:AB_2687931
Mouse monoclonal IgG	Cell Signaling Technology	Cat # 5415 RRID:AB_10829607
Rabbit polyclonal anti-α-sarcomeric	Abcam	Cat # ab137346 RRID:AB_2909405
Rabbit polyclonal anti-vimentin	Abcam	Cat # ab45939 RRID:AB_2257290
Rabbit monoclonal anti-α-smooth muscle actin	Cell Signaling Technology	Cat # 19245 RRID:AB_2734735
Alexa Fluor 488 conjugated WGA	ThermoFisher Scientific	Cat # W11261
Mouse monoclonal anti-His	ThermoFisher Scientific	Cat # MA1–21315
Mouse monoclonal anti-GST	Genscript	Cat # A00865 RRID:AB_914654
**Recombinant DNA**		
pcDNA3 *mHif2a*-P405A/P531A	Addgene	Plasmid # 18956
pcDNA3 *mHif2a*-P405A/P530V/N851A	Addgene	Plasmid # 44027
pcDNA3 *mHif2a*	Addgene	Plasmid # 18950
pLV6 *Bmal1*-luc	Addgene	Plasmid # 68833
pcDNA3 *mBmal1*	Addgene	Plasmid # 31367
pcDNA3 *mHif1a*-P402A/P577A/N813A	Addgene	Plasmid # 47334
**Chemicals**		
Nobiletin	Cayman Chemical	Cat # 15421
Polyethylene glycol (15)-hydroxy stearate	Millipore Sigma	Cat # 42966
Recombinant mouse AREG	R&D Systems	Cat # 989-AR
Dimethylallyl glycine	Cayman Chemical	Cat # 71210
2,3,5-Triphenyltetrazolium chloride	Sigma-Aldrich	Cat # T8877
Evans blue dye	Sigma-Aldrich	Cat # E2129
Paraformaldehyde	Millipore Sigma	Cat # P6148
Trypsin-EDTA	ThermoFisher Scientific	Cat #15400054
Heparin	StemCell Technologies	Cat # 07980
Anhydrous ethyl alcohol	Commercial Alcohols	Cat # P016EAAN
Hydrochloric acid (HCl)	Laboratoire Mat	Cat # CR-0166
Dimethyl sulfoxide	Millipore Sigma	Cat # D2650
Sodium Chloride (NaCl)	ThermoFisher Scientific	Cat # S271–3
Potassium chloride (KCl)	Millipore Sigma	Cat # P9541
Sodium bicarbonate (NaHCO_3_)	Millipore Sigma	Cat # S5761
Sucrose	ThermoFisher Scientific	Cat # S5–3
Triton X-100	Amresco	Cat # M143
Protein A/G resin	ThermoFisher Scientific	Cat # 53133
anti-Flag M2 beads	Millipore Sigma	Cat # A2220
Glutathione magnetic agarose beads	GE Healthcare	Cat # 17–0756-01
**Critical commercial assays**		
NE-PER^™^ Nuclear and cytoplasmic extraction kit	ThermoFisher Scientific	Cat # 78835
SimpleChIP^®^ enzymatic chromatin IP kit	Cell Signaling Technology	Cat # 9002
Mouse cardiac troponin-I SPARCL^™^ kit	Life Diagnostics	Cat # CTNI-SP-1
Click-iT^™^ Plus TUNEL assay kits	ThermoFisher Scientific	Cat # C10617
Duolink^®^ In Situ PLA^®^ Probe Anti-Rabbit PLUS	Millipore Sigma	Cat # DUO92002
Duolink^®^ In Situ PLA^®^ Probe Anti-Mouse MINUS	Millipore Sigma	Cat # DUO92004
Secrete-pair dual luminescence assay kit	GeneCopoeia	Cat # LF031
**Experimental models: Cell lines**		
HEK-293	ATCC	Cat # CRL-1573
Human Cardiac Myocytes	ScienCell	Cat # 6200
**Experimental models: organisms/strains**		
Mouse: STOCK *A1cf*^Tg[Myh6–cre/Esr1*]1Jmk^/J	Jackson	RRID:IMSR_JAX:005650
Mouse: B6.129-*Hif1a*^*tm3Rsjo*^/J	Jackson	RRID:IMSR_JAX:007561
Mouse: STOCK *Epas1*^*tm1Mcs*^/J	Jackson	RRID:IMSR_JAX:008407
Mouse: B6.129S4(Cg)-*Bmal1*^*tm1Weit*^/J	Jackson	RRID:IMSR_JAX:007668
Mouse: B6;129-*Areg*^*tm1Dle*^/Mmnc	MMRRC	MMRRC_011533-UNC
**Oligonucleotides**		
*HIF1A* siRNA	ThermoFisher Scientific	Cat # 4390825 (s6539)
*HIF2A* siRNA	ThermoFisher Scientific	Cat # 4390825 (s4698)
*BMAL1* siRNA	ThermoFisher Scientific	Cat # 4392420 (s1613)
*HIF1B* siRNA	ThermoFisher Scientific	Cat # 4392420 (s1616)
*CLOCK* siRNA	ThermoFisher Scientific	Cat # 4392420 (s18392)

## Data Availability

The accession numbers for the bulk RNA sequencing in this paper are available in NCBI GEO dataset. Mouse myocardial ischemia and reperfusion injury heart bulk RNA-seq data: GSE255307, Human surgical left ventricular bulk RNA-seq data: GSE. Cryo-EM map of the BMAL1/HIF2A/DNA complex was deposited to the EMDataBank with accession number EMD-43237. The corresponding atomic model was deposited to the RCSB Protein Data Bank with accession number 8VHG.
